# A novel explainable AI for revealing determinants of cancer drug response through integrative multi-omics analysis

**DOI:** 10.3389/fonc.2026.1759001

**Published:** 2026-05-18

**Authors:** Shynu Padinjappurathu Gopalan, Vino Sundararajan

**Affiliations:** 1 School of Computer Science Engineering and Information Systems, Vellore Institute of Technology, Vellore, India; 2 School of Bio Sciences and Technology, Vellore Institute of Technology, Vellore, India

**Keywords:** cancer drug response, explainable artificial intelligence, feature selection with metaheuristic optimization, graph attention network classifier, multi-omics drug response prediction, precision oncology, topology-aware gene sequence representation

## Abstract

**Introduction:**

Cancer drug response rates differ across patients and cell lines; however, many current computational prediction models still function as uninterpretable black boxes, offering limited insight into why a given treatment works well for some patients or cell lines but poorly for others.

**Methods:**

Here, we introduce an interpretable cancer drug response prediction framework that leverages multi-omics data from the Genomics of Drug Sensitivity in Cancer 2 (GDSC2) resource, including genomics, transcriptomics, and proteomics, where available, together with explicit chemical drug descriptors derived from SMILES and InChI representations. We use a Modified Neighbor-Joining Algorithm (MNJA) to generate topology-aware gene-sequence trees. Combined multi-omics and drug features are summarized into high-level deep descriptors via a decimal-scaled GoogLeNet (DS-GoogLeNet), together with lightweight handcrafted features. A Smoluchowski Kookaburra Optimization Algorithm (SKOA) then selects informative multimodal features, which are used to classify the sensitivity or resistance of each cell line-drug pair with an explainable Aranda Graph Attention Network (EA-GAT).

**Results:**

By analyzing model behavior using SHAP-based feature attributions and subsequently subjecting SHAP-ranked genes to pathway enrichment analysis, we highlight the recurrent involvement of the PI3K/AKT/mTOR pathway and related downstream signaling cascades in drug response. Under leakage-safe stratified 10-fold cross-validation on 2614 GDSC2 cell line-drug pairs, the framework attains an accuracy of 95.87% and an F1-score of 95.87%, with an area under the receiver operating characteristic curve (AUROC) of 0.957 and an area under the precision-recall curve (AUPRC) of 0.946.

**Discussion:**

Overall, the framework appears to predict drug response accurately while also supporting biologically meaningful interpretation, making it a useful computational tool for hypothesis generation and biomarker-focused investigation in oncology.

## 1 Introduction

Cancer remains one of the major causes of illness and death worldwide, despite substantial progress in screening and treatment (1). Drug therapy is, of course, central to the management of many human diseases, including allergies, asthma, infections, and cancer (2). To inhibit tumor proliferation and improve patient outcomes, modern oncology relies on pharmaceutical products that disrupt critical biological pathways involved in tumor cell division (e.g., DNA replication), mitotic progression, or growth factor signal transduction (3). Unfortunately, not all patients experiencing the same type of cancer, nor all tumors of the same histopathologic classification, react equally well to the same drug. While some patients may achieve considerable benefits from using the drug, other patients may receive little benefit but experience adverse reactions.

Accurately predicting how a given tumor will respond to a particular drug before treatment is therefore critical. Cancer drug response prediction (CDRP) seeks to learn the relationship between molecular profiles and observed drug sensitivity so that ineffective drugs can be avoided, unnecessary toxicity reduced, and treatment decisions aligned more closely with the biological characteristics of each patient’s disease (4, 5). Large-scale pharmacogenomic resources, such as cancer cell line panels with matched molecular and drug-response data, provide an important substrate for developing and benchmarking such predictive models.

In recent years, artificial intelligence and machine learning have become crucial in predicting cancer drug responses. The early prediction methods relied on classic machine learning approaches like support vector machines, decision trees and ensemble learning to predict the sensitivity of drugs from single-omics level such as gene expression data, while newer methodologies tend to use deep learning - based methods including reference drug-based deep neural networks that use fully connected models and CNNs to model nonlinear relationships between molecular features and drug responses (6–9). These approaches have improved predictions in many cases, but are generally constrained to using only partial amounts of available molecular information and often act as black boxes that reveal nothing about the reasons for their predictions (10).

The current research in this area has several shortcomings. First, most CDRPs use data from a single molecular modality (e.g. transcriptional data) and therefore do not fully integrate complementary information from genomics, transcriptomics, and proteomics. This partial view of tumor biology can constrain predictive power and obscure mechanisms that drive sensitivity or resistance. Second, drug molecular structure is rarely modeled by CDRPs. However, molecular structure significantly affects how drugs interact with cells (i.e., pharmacodynamics), how the drug is metabolized and excreted (i.e., pharmacokinetics); modeling structure could improve the accuracy of predictions made by CDRPs (11). Third, although some CDRP models identify genes whose expression correlates with drug response, they often provide only limited interpretability and do not support clear gene- or pathway-level explanations that can be examined biologically (12). This relationship held true throughout all levels of response as measured within this panel.

These gaps motivate the development of an integrative and explainable framework for cancer drug response prediction. In this work, we propose a model that combines multi-omics data with explicit drug molecular representations and is coupled to an Explainable AI (XAI) layer. Briefly, our model produces topology-aware sequences and trees using the MNJA (Modified Neighbour Joining Algorithm), generates a variety of attributes, uses DS-GoogLeNet to select optimized features based on a predefined criterion, and then determines cell line–drug interactions using a graph-attention classifier. We use *post-hoc* SHAP analysis and pathway enrichment on top of the predictive core to determine which genes and signaling pathways are responsible for each individual prediction. An in-depth analysis of a case study involving PI3K inhibitors demonstrates that our model can recover known predictors of response and that the PI3K-AKT/mTOR signaling pathway plays a significant role in determining response to these drugs. Therefore, our model bridges numeric predictions with interpretive biological evidence.

In summary, this study advances cancer drug response prediction by addressing both the integration and interpretability challenges. The major contributions of this study include:

- Integrative multi-omics modeling: A single framework is used to develop a representation of each cancer cell line by integrating three types of data (genomics, transcriptomics, and proteomics) which provides a more complete basis for predicting cancer drug response.
- Explicit incorporation of drug structure: Each drug’s molecular structure is encoded into the model along with its cellular properties; thus, providing a better understanding of how drugs may affect cells differently based on their unique chemical makeup.
- Topology-aware gene representation: We generate sequence- and tree-based features via MNJA to obtain more informative and structured gene-level representations.
- Rich, optimized feature extraction and selection: We extract a large, diverse set of features using attribute-based descriptors and DS-GoogLeNet.Later, followed by an optimization-guided feature selection step that favors compact, generalizable subsets.
- Explainable prediction with pathway-level insight: We employ SHAP and pathway enrichment analysis to provide gene and pathway-level explanations of model predictions. A detailed PI3K inhibitor case study demonstrating a direct link between the model’s rationale and established biological mechanisms of cancer is also provided.
- Experimental *in vitro* validation: The model’s predictive results were validated using a separate 72-hour dose-response experiment across six genomically characterized cell lines to test whether Pictilisib inhibited PI3K activity. We found that we achieved a binary accuracy of 83.3% and good agreement in ranked-order sensitivity values between our model and the measured IC_50_ values.

### 1.1 Related work

The literature is rich in research on predictive models of cancer drug response using machine learning or deep learning methods (e.g., across different modalities and architectures) that have focused on various aspects of translation. Early examples of applying deep learning to predict drug sensitivity based on gene expression were reported by Chawla et al., who paired a high-dimensional gene expression matrix with drug information and trained a deep neural network to distinguish between effective and ineffective drugs (11). These researchers demonstrated that predicting which genes will be responsive to a particular drug based on gene expression alone yields reasonable accuracy. However, they did so within a single omics dimension, and their ability to explain the predictive features they identified was limited to broad-level interpretations of feature importance and lacked methodical integration with additional molecular dimensions.

Hostallero et al. introduced TINDL (Tissue-Informed Normalisation Deep Learning) to strengthen preclinical-to-clinical transfer (12). The approach normalizes gene expression in a tissue-aware manner before training deep models to predict anti-cancer drug response in patients. By accounting for tissue context, TINDL narrows the gap between cell lines and clinical samples and surfaces candidate biomarkers along the way. The pipeline is nevertheless computationally demanding, relies almost exclusively on transcriptomics, and offers no explicit encoding of drug molecular structure or fine-grained mechanistic rationale for its predictions.

Paltun et al. approached the problem from a data integration angle with DIVERSE, a Bayesian framework that fuses gene expression, drug similarity, and protein–protein interaction information for precise drug response prediction (13). Joint modeling of these heterogeneous sources yielded clear gains over single-modality baselines, reinforcing the case for integrative learning. Yet feature selection was not explicitly optimized for generalization, and the model remained comparatively opaque: recovering clean gene- or pathway-level rationales for individual predictions is difficult.

In a different vein, Lee et al. developed a gene-centric CDRP method built around convolutional encoders (14). Here, gene expression and somatic mutation profiles are reshaped into tensor representations and passed through a CNN to predict sensitive versus resistant phenotypes. The design exploits spatial structure in the constructed tensors and effectively uses combined genomic features. Drugs, however, are largely handled as categorical labels, and detailed molecular descriptors are not deeply integrated—constraints that limit the method’s ability to disentangle drug-specific mechanisms.

Complementing these efforts, Qureshi et al. assessed machine learning-based drug response prediction in lung cancer patients using an Extreme Gradient Boosting model trained on genomics and clinical variables (15). The study made a convincing case for combining clinical context with molecular features. Its reliance on time-intensive longitudinal data collection is a practical drawback, and drug chemistry is not explicitly encoded. Interpretability, in turn, is delivered through tree-based feature importance rather than pathway-aware explanations.

Recent work has expanded the use of multi-omics data in CDRP. Wang et al. proposed a deep learning approach that integrates multiple omics layers—including gene expression, copy number variation, mutation, and protein array data—together with graph embeddings based on biological networks, using attention mechanisms to weight contributions from different omics types (16). Liu and Mei developed NDSP, which combines multi-omics data with similarity network fusion and deep learning to reduce dimensionality and mitigate overfitting, while preserving meaningful similarity structure among samples (17). Sharma et al. introduced DeepInsight-3D, which transforms multi-omics profiles into multi-channel images and applies convolutional neural networks to predict anti-cancer drug responses (18). Ahmad et al. recently applied machine learning to genomic profiling and drug discovery in lung cancer (19). These methods demonstrate that structured representations of multi-omics data can substantially improve predictive performance, but they often provide limited direct interpretability at the level of specific genes and pathways.

Other studies have explicitly integrated multi-omics features with detailed drug representations. Mohammadzadeh-Vardin et al. proposed DeepDRA, which uses autoencoders to integrate multi-omics data with drug descriptors and fingerprints in a drug repurposing context (20). Wu et al. introduced PASO, a framework that uses pathway-based difference features derived from multi-omics data and SMILES representations of drugs, combined with transformer encoders, multi-scale convolutions, and attention mechanisms to capture complex drug– cell interactions (21). These approaches highlight the advantages of representing both the cellular and drug dimensions in a richer, more mechanistic manner, but their interpretability is often limited to attention weights or global feature rankings, and proteomics is not always systematically incorporated.

Explainability has begun to receive more focused attention in this domain. Tang and Gottlieb proposed PathDSP, an explainable model that combines chemical structure fingerprints with pathway level enrichment scores derived from gene expression, mutation, and copy number variation data (22). By operating at the pathway level and applying SHAP analysis, PathDSP provides more transparent, pathway-centric explanations of drug sensitivity. Nevertheless, it does not fully exploit the breadth of available omics modalities, such as proteomics, and its explanations are primarily aggregated at the pathway level rather than at the level of individual genes and their interactions with specific drugs. Broader surveys and mapping studies on deep learning for CDRP further emphasize that many high-performing models still trade interpretability for accuracy and rarely deliver mechanistic insight that can be directly related to known signaling cascades and drug mechanisms (23).

In parallel, several multi-omics integration studies in oncology more generally, including recent work in breast cancer, have shown that AI-based integration of heterogeneous molecular data can improve precision stratification and drug resistance prediction (24).Recently, machine learning approaches have shown considerable promise in lung cancer treatment-response prediction. Interpretable frameworks using genomic and clinical variables have been proposed for drug-response analysis in non-small cell lung cancer (NSCLC) ()**?**, while integrated multi-omics transcriptomic signatures have also been validated for immunotherapy-response stratification through FOXOmediated transcriptional features ()**?**. Taken together, these studies reinforce the importance of jointly modeling multiple omics layers and suggest that the same principles can be extended to drug-response prediction in a more mechanistically grounded way (10).

Taken together, the literature points to two broad conclusions: deep learning combined with multi-omics integration can deliver strong performance on cancer drug response prediction, and performance generally benefits from the inclusion of drug molecular information and biological priors such as pathways and network structure. Three critical gaps nevertheless persist. Many frameworks still privilege a subset of omics modalities, or combine them only loosely, rather than constructing a genuinely unified multi-omics representation. Detailed drug molecular structure is not consistently encoded alongside cellular features in ways that support mechanistic interpretation. And interpretability is too often reduced to generic feature-importance scores or attention maps; relatively few models are built to yield coherent gene- and pathway-level explanations that can be cross-checked against established cancer biology.

The framework proposed here is designed to close these gaps. It integrates genomics, transcriptomics, and proteomics with explicit drug structural representations, and it treats explainability as a core design principle rather than an afterthought—using SHAP-based attributions and pathway enrichment analyses to expose the mechanistic determinants of drug response.

The rest of the paper is organized as follows. Section 2 describes the materials and methods, including data processing, feature construction, model architecture, and evaluation protocol. Section 3 presents the experimental results and the associated biological interpretation. Section 4 discusses the findings in the context of existing work and outlines the limitations of the study. Finally, Section 5 concludes the paper and highlights directions for future development.

## 2 Materials and methods

### 2.1 Data source and cohort curation

The primary pharmacogenomic data source for our investigation was the Genomics of Drug Sensitivity in Cancer 2 (GDSC2) (25). The GDSC2 database contains virtually all of the drug sensitivity measures for nearly 700 cancer cell lines and for over 138 different anti-cancer drugs across an estimated 75,000 experiments. We did not use the complete original GDSC resource. Rather, we created a cohort of matched cell line–drug pairings to be used in our analysis for which both response measures, molecular characteristics, and structural characteristics of each drug were available.

Our starting cohort had 8,146 cell line–drugs. From this cohort, we deleted 854 pairs based on missing or invalid responses. An additional 4,179 pairs were removed due to insufficient information about their molecular profiles. Lastly, another 499 pairs were removed from the cohort since no drug structural information was available. Thus, there remained a total of 2,614 labeled cell line–drug pairs in the cohort to be analyzed. **Table 1** summarizes all aspects of the data curation process.

**Table 1:** Summary of the data curation process used to derive the final analytical cohort from the matched GDSC2 cell line–drug pairs.

Curation step	Retained pairs	Excluded at this step	Reason
Initial matched candidate cohort	8,146	–	Candidate cell line–drug pairs after matching core response, molecular, and drug-structure fields
After removing missing/invalid response measurements	7,292	854	No usable IC_50_ or invalid response summary
After removing incomplete molecular profiles	3,113	4,179	Missing required molecular feature information for the selected omics feature set
After removing pairs without usable drug structural information	2,614	499	Missing or unusable structural representation fields
Final labeled	**2,614**	**5,532**	Used in all experiments

Bold values indicate the final labeled analytical cohort used in all experiments.

All subsequent preprocessing, feature extraction, feature selection, and model training steps were carried out within the training folds of the stratified 10-fold cross-validation procedure. The same curated cohort was used throughout the comparative experiments reported in this study.

### 2.2 Response label definition

Cancer drug response was formulated as a binary classification task. Each retained cell line– drug pair was assigned a sensitivity label using an IC_50_-based threshold. Pairs with IC_50_ ¡ 1 µM were labeled as sensitive, whereas pairs with IC_50_ *≥* 1 µM were labeled as resistant. Applying this threshold to the final curated cohort of 2,614 cell line–drug pairs yielded 1,241 sensitive pairs (47.48%) and 1,373 resistant pairs (52.52%). The same threshold was used in the orthogonal Pictilisib validation analysis to maintain consistency between the computational and experimental evaluations. Stratified 10-fold cross-validation was used to preserve class proportions across folds during training and testing.

### 2.3 Proposed cancer drug response prediction methodology

The proposed work performs Cancer Drug Response Prediction (CDRP) based on the Smoluchowski.

**Figure 1:**
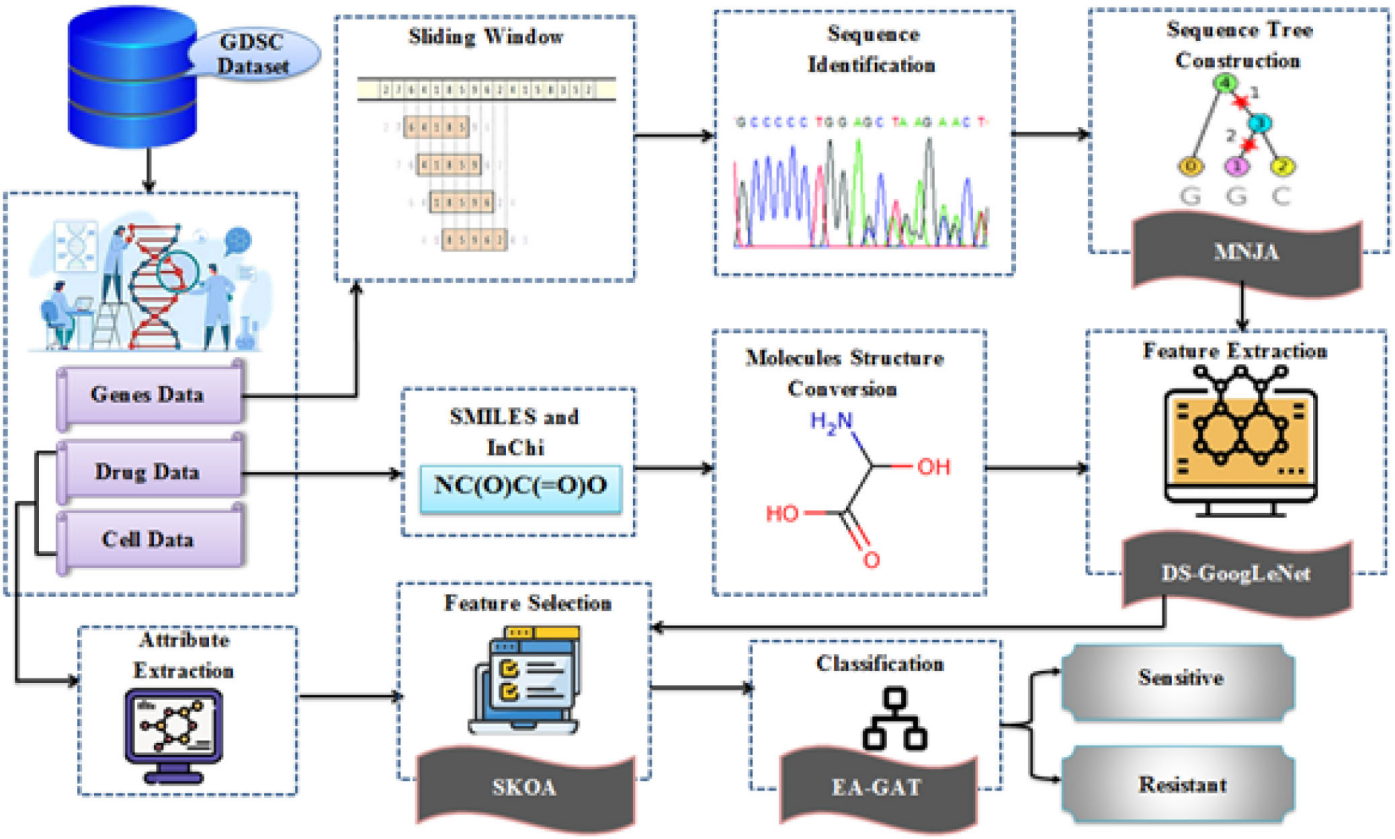
Architecture of the proposed cancer drug response prediction model.

Kookaburra Optimization Algorithm (SKOA) and an Enhanced Graph Attention Network (EAGAT). The overall framework integrates multi-omics information from cancer cell lines with explicit drug molecular structure and an explainability layer based on SHAP and pathway enrichment. **Figure 1** depicts the architecture of the proposed model.

#### 2.3.1 Input data

The curated GDSC2 cohort described in Sections 2.1 and 2.2 was used to define three main entities in this study: cancer cell lines, genes, and drugs, represented as shown in Equations (1–3)

(1) $$C=\{c_{i}|i=1,\ldots ,N_{c}\},$$

(2) $$G=\{g_{k}|k=1,\ldots ,N_{g}\},$$

(3) $$D=\{d_{j}|j=1,\ldots ,N_{d}\},$$

where *N_c_*, *N_g_*, and *N_d_* denote the number of cell lines, genes, and drugs under study, respectively.

For each cell line *c_i_* ∈ *C*, we extracted genomics and transcriptomics features, including binary mutation calls, copy number variation, and normalized gene expression values. Where protein or phospho-protein measurements were available within the matched molecular profile, these were incorporated as additional covariates to approximate proteomic activity. All continuous features were standardized to zero mean and unit variance within the training folds, as described in the evaluation protocol.

For each drug *d_j_ ∈ D*, we collected the corresponding response summaries together with structural encodings such as SMILES and InChI strings, along with metadata including drug id, name, and annotated targets. Binary sensitivity labels for each cell line–drug pair were assigned as described in Section 2.2. These components form the basis of the integrative multi-omics and structural representation used in the proposed framework.

#### 2.3.2 Sliding window

For efficient Sequence Identification (SI), a sliding window is generated for each gene sequence. Let *s_k_* denote the sequence associated with gene *g_k_*, let *L_k_* denote the length of *s_k_*, let *L_w_* denote the window length, and let *S_w_* denote the step size. The set of windows for *g_k_* is defined as shown in Equation (4)

(4) $$W_{k}=\left\{s_{k}\left[p:p+L_{w}-1\right]|p=1+mS_{w},\;m=0,1,\ldots ,\left[\frac{L_{k}-L_{w}}{S_{w}}\right]\right\},$$

where each window spans *L_w_* consecutive positions of the sequence. In this way, each gene sequence is partitioned into overlapping or non-overlapping windows, depending on the value of *S_w_*, and the resulting windows are passed to the next stage for sequence identification.

#### 2.3.3 Sequence identification

In this step, Sequence Identification (SI) is applied to the set of sliding-window segments *W_k_* derived for each gene *g_k_*. The purpose of SI is not to infer biological evolution, but to convert each window into a structured segment representation that can later be organized into the tree-based representation described in Section 2.3.4. For each window, the local ordering and repetition pattern of the encoded sequence symbols are examined to determine whether the window contains one of a small set of predefined structural-event patterns.

The event categories considered in this study are insertion, deletion, inversion, mirror, and duplication. An insertion indicates the appearance of an additional short subsequence within the current window relative to the local reference ordering; a deletion indicates the absence of an expected subsequence; an inversion indicates that a local subsequence appears in reversed order; a mirror event indicates a reflected local arrangement pattern; and a duplication indicates repetition of a subsequence within the same windowed region.

Following the above pattern verification, each analyzed window is represented as a detected segment and associated with its corresponding event label. Thus, for each gene *g_k_*, the SI stage outputs a collection of descriptor-ready segment units of the form as shown in Equation (5)

(5) $$S_{k}={\left\{\left(s_{k,r},\;e_{k,r}\right)\right\}}_{r=1}^{n_{k}},$$

where *s_k,r_* denotes the *r*th detected segment for gene *g_k_*, *e_k,r_* denotes its corresponding event label, and *n_k_* is the number of detected segments obtained from the windows of *g_k_*. These segment–event pairs form the direct input to the descriptor-based dissimilarity calculation and Sequence Tree construction stage described next.

#### 2.3.4 Sequence tree construction

By utilizing the Modified Neighbor-Joining Algorithm (MNJA), the Sequence Tree (ST)—from which gene features are extracted—is constructed for the genes in *G*. For ST construction, the classical Neighbor Joining Algorithm (NJA) is used as the basis. In the present framework, NJA is adapted as a computational procedure for organizing the detected sequence segments and their pairwise relationships into a structured tree representation. It is not used here for phylogenetic or evolutionary inference.

For each gene, the identified segments are represented using descriptors that are used to compute a pair-wise dissimilarity matrix. This matrix serves as input to MNJA’s iterative merging of similar segments to construct, step by step, a tree structure (i.e., an ordered graph). The final constructed tree provides a topological-computational model that describes both the sequences’ local patterns as well as the inter-relationship among all event types detected.

The tree structure can be written as shown in Equation (6)

(6) $$T_{g}=\left(V_{g},E_{g}\right),$$

where *V_g_* indicates the set of nodes (segments) for gene *g*and *E_g_* indicates the set of branches. The branch lengths ℓ(u,v) between segment representations *u*and *v*are estimated using their descriptor-based dissimilarity as shown in Equation (7)

(7) ℓ(u,v)=d(u,v),

where *d*(*·,·*) denotes the descriptor-based dissimilarity function defined over segment representations.

**Figure 2:**
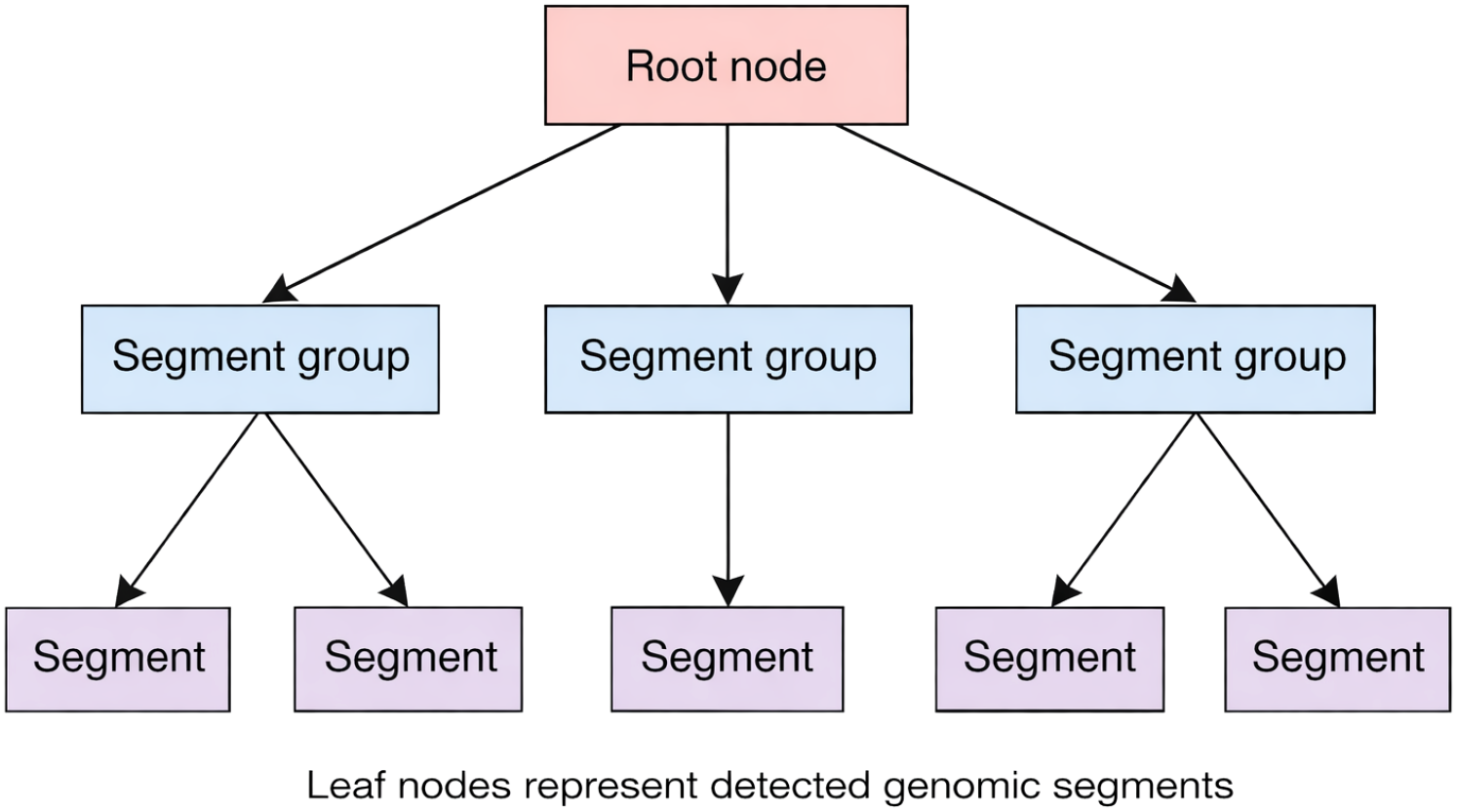
Illustrative sequence tree constructed from detected genomic segments and their hierarchical grouping. Internal nodes represent descriptor-based segment groupings, and leaf nodes correspond to detected segments used for downstream feature extraction.

Thus, the tree *T_g_* is constructed based on the dissimilarity matrix and the estimated branch lengths, as shown in **Figure 2**.

When compared to commonly employed “bag-of-mutations” or other flat gene-level representations, MNJA enables the maintenance of structural relationships (i.e., topological structure) among the detected genomic segments and the events they represent, allowing downstream feature extractors to use topology-aware patterns rather than independent binary indicators. The features are extracted from *T_g_* as explained in Section 2.3.6.

#### 2.3.5 Molecules structure conversion

**Figure 3:**
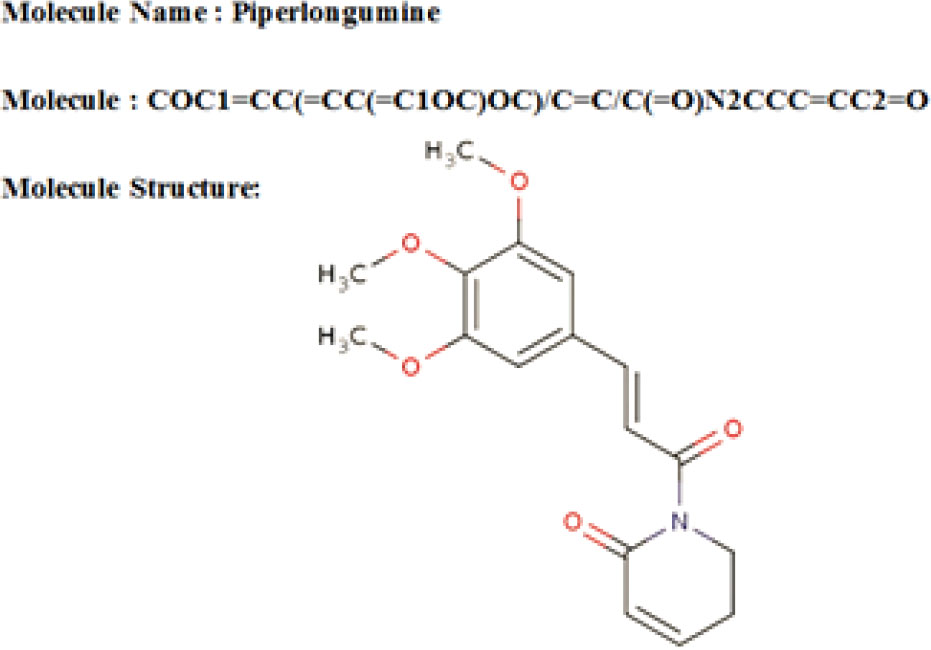
Example of drug molecule structure representation.

Meanwhile, the SMILES and InChI strings representing the drug molecules are collected from the drug set *D* and converted into a structured representation *M_j_* for downstream feature extraction. For each drug *d_j_ ∈ D*, *M_j_* captures the molecular structure in two complementary forms: a molecular graph describing atom-level connectivity and a standardized two-dimensional depiction used for image-based representation, as illustrated in **Figure 3**.

This conversion provides a consistent structural input for both handcrafted descriptor extraction and deep feature learning in the subsequent stage. In this way, the framework treats each drug as a structured entity whose geometry and connectivity can be used during feature extraction.

#### 2.3.6 Feature extraction

We first extract descriptive features from both the sequence trees *T_g_* and the molecular structures *M_j_*. The sequence-tree component provides structural information derived from the detected sequence motifs and events, while the molecular component encodes structural characteristics derived from the corresponding drug compounds. Together, these two components form the input used for downstream feature learning.

For DS-GoogLeNet input construction, each matched cell line–drug pair was represented as a fixed-size three-channel tensor. The sequence-tree representation derived from *T_g_* was first encoded as a two-dimensional structured map. This map preserved the detected segment relationships and event patterns. The drug representation derived from *M_j_* was expressed separately as a standardized two-dimensional depiction. To maintain structural consistency, the sequence-tree maps were standardized using zero-padding, whereas the drug depictions were resized to a common spatial resolution. The resulting representations were then aligned to a shared size of 224 *×* 224 and fused along the channel dimension to form a composite tensor $X\in {\mathbb{R}}^{224\times 224\times 3}$. Continuous-valued channels were normalized within the training folds, while binary event indicators were retained in encoded form. This channel-wise fusion enabled DS-GoogLeNet to jointly analyze cellular and drug representations while preserving their complementary structural information.

Features are learned from the sequence trees using DS-GoogLeNet. In addition, handcrafted descriptors are used to capture statistical and texture-related information. From the molecular structures, features such as Gray-Level Co-occurrence Matrix (GLCM), texture descriptors, Local Tetra Pattern, and Local Binary Pattern (LBP) are extracted, together with the deep features produced by DS-GoogLeNet.

Standard GoogLeNet is adopted as the base feature extraction model because the fused representation of each cell line–drug pair is expressed as a structured three-channel tensor in which local spatial neighborhoods preserve segment-event arrangements from the sequence tree together with the standardized drug depiction. Under this tensorized representation, convolutional filtering becomes appropriate for learning joint local patterns across the cellular and drug channels. GoogLeNet was selected because its multi-scale convolutional blocks can capture patterns at different receptive fields within this fused representation, which is useful when informative structures occur at different spatial granularities.

In the proposed method, DS-GoogLeNet is used as the deep feature learner for the hybrid representation obtained from *T_g_* and *M_j_*. Here, decimal scaling is applied as a numerical range control step on the convolutional responses before non-linear activation, so that feature magnitudes from the fused channels remain on a comparable scale during feature extraction. Let the tensor formed by combining the sequence-tree and molecular-structure representations be denoted by *X*.

The tensor *X* is first convolved to produce the pre-activation response map (Equation (8))

(8) Z=W∗X+b,

after which a decimal-scaling operation is applied to *Z* before the ReLU transformation. The resulting activated feature map is written as (Equation (9))

(9) $$H=\phi_{\text{ReLU}}\;\left({\text{Ψ}}_{\text{DS}}\left(Z\right)\right),$$

where ${\text{Ψ}}_{\text{DS}}\left(\cdot \right)$ denotes decimal-scaling normalization of the convolutional responses and $\phi_{\text{ReLU}}\;\left(\cdot \right)$ denotes the Rectified Linear Unit activation.

The resulting feature map is then down-sampled with weight value *W_d_* and passed through a fully connected layer f(·) as (Equations (10, 11))

(10) $$H_{d}=\text{Pool}\left(H;W_{d}\right),$$

(11) $$O=f\left(H_{d}\right),$$

where the pooling operation reduces the spatial resolution and *O* denotes the high-level feature vector. During pretraining, the output is activated using the softmax function as (Equation (12))

(12) P(y|X)=softmax(O),

where P(y|X) represents the predicted class probabilities. For the integrated framework, the penultimate-layer representation is used as the extracted deep feature vector.

In addition to these deep features, handcrafted descriptors including GLCM, texture, Local Tetra Pattern, and LBP features are computed from *T_g_* and *M_j_* and concatenated with the DS-GoogLeNet features to form the complete feature representation used in the subsequent stages.

#### 2.3.7 Attributes extraction

Beyond the structural features extracted from the sequence trees and drug molecular representations, we retain a set of auxiliary annotation variables for each matched cell line–drug pair. On the cell-line side, these attributes are drawn from the matched annotation metadata and include lineage or primary disease category, molecular subtype, where available, and other matched line-level metadata that help contextualize the omics profile. On the drug side, we retain identifier and annotation fields including drug id, drug name, annotated targets, and the corresponding SMILES and InChI representations.

The DS-GoogLeNet-based feature extraction procedure is summarized in Algorithm 1.

Algorithm 1

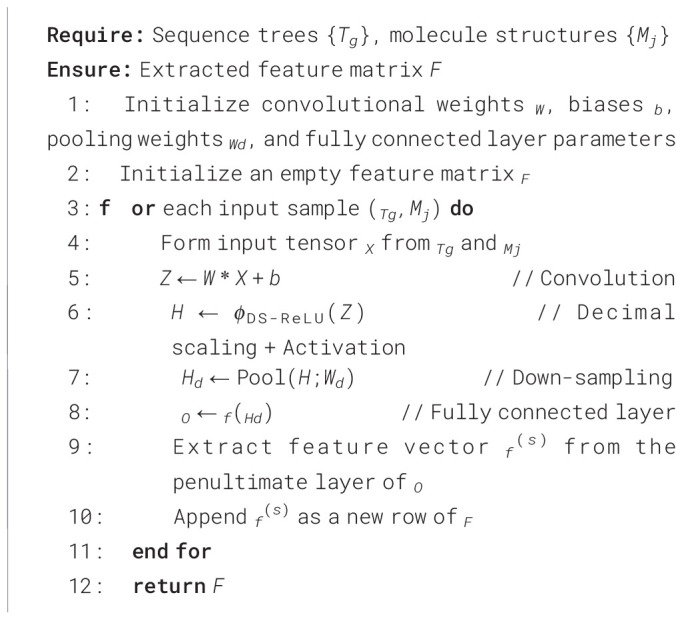

After suitable preprocessing and numerical encoding, these auxiliary variables are organized into an attribute matrix *A* and concatenated with the extracted feature matrix *F* to form the full representation as shown in Equation (13)

(13) $$X_{\text{full}}=\left[F∥A\right],$$

where [·∥·] denotes concatenation. This combined representation integrates learned structural features with contextual annotation variables and is then passed to the feature-selection stage.

#### 2.3.8 Feature selection

The feature vector *X*_full_ is formed by combining omics features, drug structural features, and auxiliary attributes, resulting in a heterogeneous representation. To retain the most informative subset before classification, a feature-selection stage is applied. In this work, we employ the Smoluchowski Kookaburra Optimization Algorithm (SKOA), an enhanced variant of the Kookaburra Optimization Algorithm (KOA) that incorporates a Smoluchowski-guided search mechanism. Rather than using the full feature set directly, SKOA searches for a smaller group of features that still captures the patterns most useful for cancer drug response prediction. The full representation *X*_full_ initially contained 2,378 candidate features. During stratified 10-fold cross-validation, SKOA retained an average of 244.6 ± 5.9 features per fold, with a median of 244 features. This means that the selected subset accounted for 10.29% of the original feature space. The consistency of the selected subsets across folds was assessed using the pairwise Jaccard index, which gave a mean stability of 0.69. The Smoluchowski-guided component is incorporated to improve the search process and reduce the tendency of KOA to become trapped in local optima. The optimal subset obtained from SKOA is then provided to the EA-GAT classifier described in the next subsection.

##### 2.3.8.1 Initialization

The population matrix *E* and the initial positions *e_g,c_* of the Kookaburras are defined as (Equation (14))

(14) $$E=\left[\begin{array}{c} E_{1}\\ \vdots \\ E_{g}\\ \vdots \\ E_{a} \end{array}\right]=\left[\begin{array}{ccccc} e_{1,1} & \cdots & e_{1,c} & \cdots & e_{1,k}\\ \vdots & \ddots & \vdots & \ddots & \vdots \\ e_{g,1} & \cdots & e_{g,c} & \cdots & e_{g,k}\\ \vdots & \ddots & \vdots & \ddots & \vdots \\ e_{a,1} & \cdots & e_{a,c} & \cdots & e_{a,k} \end{array}\right],$$

where each row *E_g_* corresponds to the *g*th Kookaburra and each column index *c* = 1*,…,k* corresponds to one decision variable.

The initial position of each element *e_g,c_* is given by Equation (15)

(15) $$e_{g,c}=\left[\left(ub_{c}-lb_{c}\right)\alpha \right]+lb_{c},$$

where *a* specifies the number of Kookaburras (population size), *k* denotes the number of decision variables (feature dimensions), α~U(0,1) is a random number, and (*lb_c,_ ub_c_*) are the lower and upper bound values for the *c*th dimension of the search space.

For notational convenience, we also denote the *g*th row *E_g_* as a candidate vector *x_g_*, and write the population as $P=\left\{x_{i}|i=1,\ldots ,N_{K}\right\}$ with *N_K_*= *a*. In vector form, the initialization in (15) can be compactly expressed as Equation (16)

(16) $$x_{i}^{\left(0\right)}=L+r_{i}⊙\left(U-L\right),$$

where *r_i_* collects the random values *α* for all *k*dimensions.

##### 2.3.8.2 Fitness

The fitness function, which updates the position of Kookaburras, is derived based on maximum classification accuracy as Equation (17)

(17) $$\text{Fit}\left(x_{i}\right)=\text{Acc}\left(x_{i}\right),$$

where $\text{Acc}\left(x_{i}\right)$ denotes the accuracy obtained using the feature subset encoded by **x***_i_*.

##### 2.3.8.3 Position updation

The position update is affected by the random selection of prey. Thus, the Smoluchowski approach that selects the suitable prey *p* is given by (Equation (18))

(18) $$p=S\left(x_{i}\right),$$

where S(·) denotes the Smoluchowski-based selection function.

*Exploration phase*: In this phase, the prey is attacked by Kookaburras, leading to a detailed search in the space. The new position of Kookaburra $x_{i}^{\text{new}}$ with constant *C*_1_ is expressed by (Equation (19))

(19) $${\text{x}}_{\text{i}}^{\text{new}}={\text{x}}_{i}^{\left(t\right)}+C_{1}\cdot \left(\text{P}-{\text{x}}_{i}^{\left(t\right)}\right),$$

where *t* denotes the current iteration.

*Exploitation phase*: Then, Kookaburra carries the prey and kills it. The new position of Kookaburra is expressed by (Equation (20))

(20) $$x_{i}^{\text{new}}=p+C_{2}\cdot \left(x_{i}^{\left(t\right)}-p\right),$$

where *C*_2_ is a constant and *t* signifies the iteration value with maximum iteration *T*_max_. Hence, the best position (optimal feature subset) x^*^ is attained.

The complete SKOA-based feature-selection procedure is summarized in Algorithm 2.

Algorithm 2

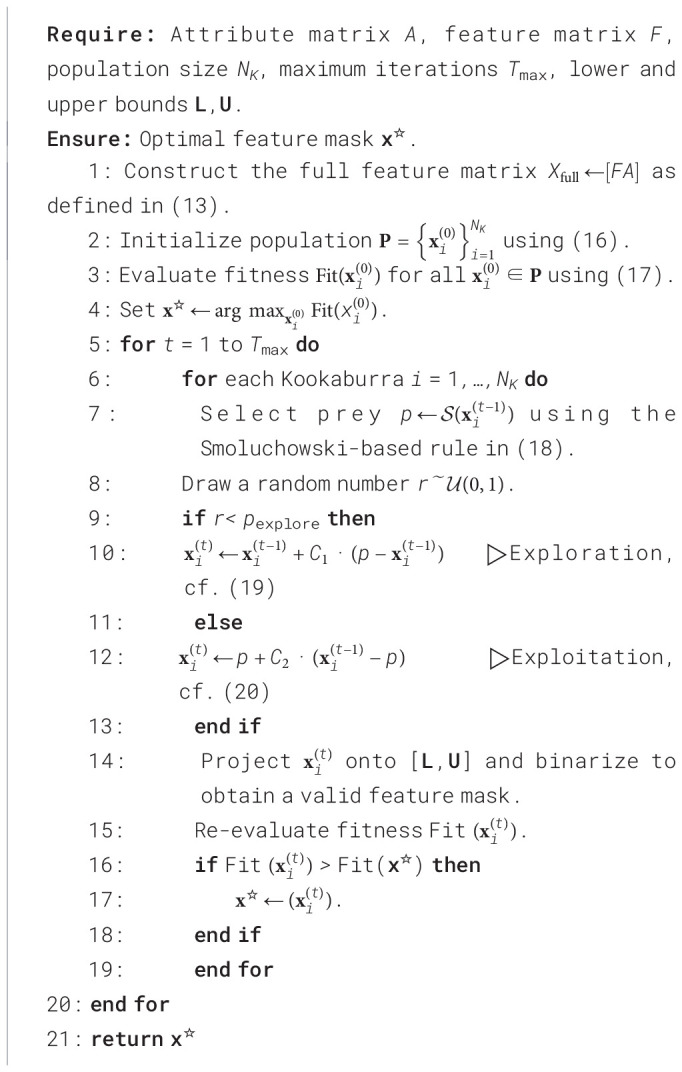

In contrast to simple filter-based or L_1_-regularized feature selection, SKOA optimizes feature subsets directly with respect to classifier performance and can capture higher-order interactions between heterogeneous features (omics, structure, attributes), which is critical in the high-dimensional, multimodal CDRP setting. The optimal feature set obtained from SKOA is then used for classification.

#### 2.3.9 Classification

**Figure 4:**
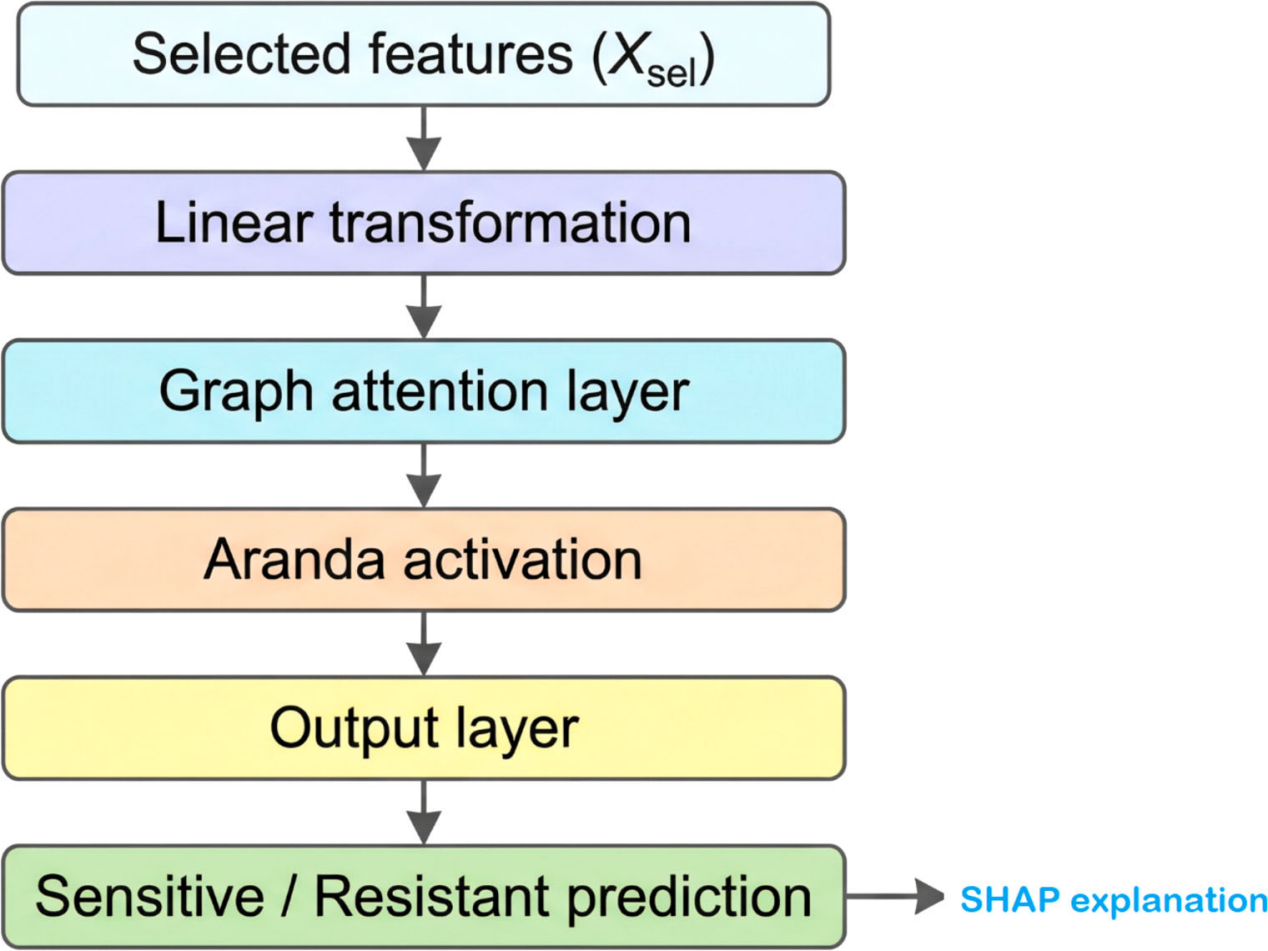
Simplified architecture of the Enhanced Aranda Graph Attention Network (EA-GAT) classifier. The selected feature vector is linearly transformed, processed through a graph-attention layer with Aranda activation, and mapped to the final drug response class. A *post-hoc* SHAP analysis stage is used to interpret feature contributions.

Classification was conducted using the optimized feature set acquired by SKOA, incorporating the Enhanced Aranda Graph Attention Network (EA-GAT). This classifier uses self-attention to capture dependencies among the selected features. In the proposed framework, the Aranda activation function is used as the non-linear transformation applied after neighborhood aggregation within the Graph Attention Network (GAT), resulting in the EA-GAT model. Its inclusion is motivated empirically in this study: as shown later in the ablation analysis, replacing it with a conventional activation leads to a modest but consistent reduction in predictive performance. The general architecture of this classifier is shown in **Figure 4**. A learnable linear transformation is applied to the input feature vector before it is passed to the attention mechanism.

Primarily, the selected feature vector is linearly transformed with the learnable weight *W_g_* and bias value *b_g_* as (Equation (21))

(21) $$H^{\left(0\right)}=W_{g}X_{\text{sel}}+b_{g},$$

where *X*_sel_ denotes the selected features.

Next, to quantify the relative importance of neighboring features, the attention score between node *u* and its neighbors is normalized as (Equation (22))

(22) $$\alpha_{uv}={\text{softmax}}_{v}\left(\sigma \left(a^{⊤}\left[H_{u}^{\left(0\right)}∥H_{v}^{\left(0\right)}\right]\right)\right),$$

where *α_uv_* signifies the self-attention mechanism output, a denotes the attention weight vector, σ(·) is a non-linear activation, and [·∥·] depicts concatenation.

The aggregated representation is then transformed using the Aranda activation function as the non-linear mapping in the graph-update step, and the resulting classifier output is subsequently interpreted using SHapley Additive exPlanations (SHAP), as follows (Equations (23–25)):

(23) $$H_{u}^{\left(1\right)}=\varphi_{\text{Aranda}}\left(\underset{v\in N\left(u\right)}{\sum }\alpha_{uv}H_{v}^{\left(0\right)}\right),$$

(24) $$\hat{y}=\text{softmax}\left(W_{o}H^{\left(1\right)}+b_{o}\right),$$

(25) $$\text{SHAP}{\left(f,X_{\text{sel}}\right)}_{j}=\phi_{j},$$

where $\hat{y}$ is the final classified output, *W_o_* and *b_o_* are the output-layer parameters, and *ϕ_j_* denotes the SHAP value associated with the feature *j* for the EA-GAT model *f*. The sensitive class is depicted as *y* = 1, and the resistant class is indicated as *y* = 0.

EA-GAT was selected over conventional multilayer perceptrons or CNNs because it can explicitly model feature dependencies (e.g., genes within a pathway or related structural descriptors of a drug) via attention on a feature graph, thereby improving both predictive capacity and interpretability.

In practice, we use a model-agnostic Kernel SHAP implementation applied to the trained EA-GAT classifier on the selected feature space. For each drug or drug class, SHAP values are estimated using a background sample of a few hundred randomly selected cell line–drug pairs from the training folds, and explanations are computed for all test samples. For global importance, SHAP values are aggregated across samples and ranked by their absolute mean value, yielding a list of influential features for each drug or drug class.

Thus, the proposed framework predicts the response of cancer drugs while also providing feature-level explanations for each prediction through SHAP values.

#### 2.3.10 Evaluation protocol

To rigorously validate the proposed framework and avoid information leakage, we employ stratified 10-fold cross-validation on the curated GDSC2 cohort. The set of all cell line–drug pairs is partitioned into ten folds with approximately equal class proportions (sensitive and resistant). In each run, nine folds are used for training, and one fold is held out for testing.

All operations that can leak information—Sliding Window segmentation, Sequence Identification, MNJA tree construction, DS-GoogLeNet feature extraction, attribute encoding, SKOA feature selection, and EA-GAT training—are fitted *only* on the training data of each fold and then applied to the held-out test fold. Stratification ensures that the class distribution is approximately balanced across folds; No additional oversampling or class weighting is applied unless otherwise stated.

Performance is measured using accuracy, precision, recall, F1-score, area under the receiver operating characteristic curve (AUROC), and area under the precision–recall curve (AUPRC), as well as calibration measures where relevant. The reported values correspond to the mean results across the ten folds, together with their standard deviations.

#### 2.3.11 Ablation studies

To assess the contribution of each major component of the framework, ablation studies are carried out under the same cross-validation protocol. In particular, we consider:

- Without SKOA: All features are used without feature selection to evaluate the importance of optimization-based feature subset selection.
- Without MNJA: Sequence Trees are not constructed, and features derived from MNJA are removed, isolating the effect of topology-aware sequence modeling.
- Without drug structural features: The model is trained using only omics features and attributes, excluding molecular structure descriptors, to assess the role of explicit chemistry.
- Non-graph classifier: EA-GAT is replaced with a standard multilayer perceptron or CNN baseline, preserving the same features but eliminating graph attention.
- Without Aranda activation: A conventional activation (ReLU) is used instead of the Aranda function to measure its impact on convergence and performance.

The results of these variants are compared with the full model to demonstrate how each component contributes to predictive performance, stability, and interpretability.

#### 2.3.12 Pathway enrichment and biological interpretation

Finally, the SHAP values computed for the trained EA-GAT model are used to identify the most influential gene-level features for each drug or drug class. Genes with high SHAP values are mapped to curated pathway databases such as KEGG, Reactome, or Gene Ontology (Biological Process). For a given drug or drug class, we:

1. Select the top *K* genes by aggregated SHAP value (typically *K* = 50).
2. Perform over-representation analysis using a standard enrichment tool (e.g., clusterProfiler or g:Profiler) against KEGG, Reactome, and Gene Ontology.
3. Adjust *p*-values for multiple testing using the Benjamini–Hochberg procedure and consider pathways with false discovery rate (FDR)< 0.05 as significantly enriched.

In particular, for PI3K inhibitors, we examine the genes with the largest positive SHAP contributions to sensitivity and resistance and perform pathway enrichment on this set. The resulting enriched pathways—including PI3K/AKT/mTOR signaling and related cascades—provide a mechanistic interpretation of the model’s predictions and demonstrate alignment between the learned determinants of drug response and established cancer biology.

### 2.4 *In vitro* validation with Pictilisib

The primary evaluation method of EA-GAT was by a stratified 10-fold cross-validation using the GDSC2 dataset. This is a very reliable way to test how well a prediction model performs while avoiding potential data leaks. Any predictions made based on publicly available cell lines require additional supporting evidence to be verified. To this end, we conducted an orthogonal wet-lab assay to determine whether the EA-GAT-predicted responses were similar to those measured in the experiments. Pictilisib (GDC-0941) was selected for testing due to its target of PI3K signaling and because SHAP had consistently identified the PI3K/AKT/mTOR signaling pathway as the most significant contributor to response (Sections 3.4 and 3.5). The goal was twofold: confirm that the EA-GAT predictions correspond to genuine biological differences in the drug response, and verify that the mechanistic interpretation derived from SHAP aligns with the known pharmacology of the PI3K inhibitor.

#### 2.4.1 Cell line selection and culture

Six cell lines from the GDSC2 resource were selected to represent diverse PI3K pathway contexts. MCF7, T-47D, and BT-474—all breast carcinoma lines carrying activating PIK3CA mutations (E545K, H1047R, and K111N, respectively)—were expected to show sensitivity owing to PI3K pathway dependence. BT-474 also harbors HER2 amplification, introducing a secondary signaling axis. HCC1954, another breast line, combines PIK3CA mutation with strong HER2 amplification and was anticipated to exhibit intermediate or borderline response. MDA-MB-231, a triple-negative breast cancer line with PTEN loss but intact PIK3CA, and A549, a lung carcinoma line driven by KRAS G12S mutation, were expected to be relatively resistant because of active bypass pathways. This panel thus spans the biological spectrum from clear PI3K dependence to pathway-independent resistance.

All lines were obtained from ATCC (Manassas, VA) and cultured under recommended conditions at 37 °C in humidified 5% CO_2_ atmosphere. MCF7, T-47D, and HCC1954 were grown in RPMI-1640 with 10% FBS and 1% penicillin-streptomycin; BT-474 in DMEM/F12 with the same supplements; MDA-MB-231 in DMEM; and A549 in F-12K medium. Routine mycoplasma testing and STR authentication were performed to ensure cell line integrity.

#### 2.4.2 Dose–response experiments

Pictilisib was sourced from Selleckchem (Houston, TX) as powder, dissolved to 10 mM in DMSO, and stored at −20 °C in single-use aliquots. On the day of treatment, working dilutions were prepared in culture medium to achieve a final DMSO concentration of 0.1% across all wells, including vehicle controls, thereby eliminating solvent artifacts.

Cells were seeded in white 96-well plates (Corning) at densities chosen to keep cultures in log-phase growth over the 72 h assay window: 2,500 cells/well for MCF7, T-47D, and HCC1954; 3,500 for BT-474; 2,000 for MDA-MB-231; and 3,000 for A549. After overnight attachment, the medium was aspirated and replaced with either drug-containing medium or vehicle. Seven concentrations across the 0.01–10 µM range were tested (0.01, 0.032, 0.1, 0.32, 1.0, 3.2, and 10 µM), each in technical triplicate. Three independent biological replicates were performed on separate days, each using cells from a different passage number.

Viability was measured at 72 h with CellTiter-Glo (Promega, Madison, WI). Plates were first equilibrated to room temperature, the reagent was added at a 1:1 ratio, and the plates were shaken for 2 min to lyse the cells. Luminescence was recorded after a 10 min stabilization period with a 0.5 s integration time per well. Signal from cell-free wells was treated as background and subtracted from all readings.

#### 2.4.3 Curve fitting and endpoint calculation

Luminescence values were normalized to the mean of vehicle-treated wells on each plate, yielding percent viability. We fit four-parameter logistic (4PL) curves to the dose–response data, as shown in Equation (26):

(26) $$v\left(c\right)=B+\frac{T-B}{1+{\left(\frac{c}{IC_{50}}\right)}^{h}},$$

where *v*(*c*) is viability at concentration *c*, *T* and *B* are top and bottom asymptotes (fixed at *T* = 100, 0 *≤ B ≤* 100), IC_50_ is the half-maximal inhibitory concentration, and *h* is the Hill slope. Fitting was done in R 4.3 using the drc package (drm function, LL.4 model), which applies nonlinear least squares. IC_50_ estimates were extracted for each replicate and averaged. We also computed normalized area under the curve (AUC) by trapezoidal integration, scaled so that AUC = 1 indicates complete resistance and AUC = 0 indicates full sensitivity.

Most fits achieved *R*^2^ *>* 0.95. One T-47D replicate had *R*^2^ = 0.93 due to scatter around mid-range doses, but its IC_50_ (0.39 µM) matched the other two replicates (0.41 and 0.43 µM), so it was retained rather than discarded.

#### 2.4.4 Agreement with model predictions

EA-GAT predictions of Pictilisib based on this line set were derived from the EA-GAT’s original cross-validation run. Each of the six lines was a member of a separate test fold because the EA-GAT had never been trained on any of the Pictilisib labels contained within them. Thus, they retained an independent validation. These EA-GAT predictions were made by assigning a binary experimental label to each of the lines in accordance with the same IC_50_ ¡ 1 µM threshold used to assign a label to each of the lines in the GDSC2 dataset; we then identified whether or not each prediction corresponded with its associated label. The standard evaluation metrics (i.e., accuracy, sensitivity, specificity, positive predictive value [PPV], negative predictive value [NPV] and Matthews correlation coefficient) were computed.

For rank-order agreement, we correlated EA-GAT sensitivity scores (continuous probabilities) with IC_50_ values using Spearman’s *ρ_s_*. High scores should correspond to low IC_50_ (strong sensitivity), so a negative correlation was expected. A second correlation was run with normalized AUC. We also tested whether IC_50_ differed between predicted-sensitive and predicted-resistant groups via Mann–Whitney *U* test. Statistical tests were performed in R and Python (scipy.stats); *p<* 0.05 was deemed significant. This validation does not re-assess overall model performance—that was established through cross-validation on 2614 pairs—but rather provides orthogonal biological evidence that predictions reflect real drug-response differences.

## 3 Results

### 3.1 Cohort summary

The final curated GDSC2 cohort used in this study comprised 2,614 labeled cell line–drug pairs after exclusion of entries with missing or invalid response measurements, incomplete molecular profiles, or unavailable drug structural information. The full cohort curation process is summarized in **Table 1**. Using the IC_50_ threshold defined in Section 2.2, the cohort comprised 1,241 sensitive and 1,373 resistant cell line–drug pairs, corresponding to 47.48% and 52.52% of the final analytical set, respectively. All methods were evaluated on this same curated cohort under an identical stratified 10-fold cross-validation protocol to ensure fair comparison across models.

### 3.2 Overall predictive performance and computational behavior of the proposed framework

The proposed framework was evaluated on the curated GDSC2 cohort described in Sections 2.1 and 2.2. The same stratified 10-fold cross-validation procedure was applied to all models. To ensure a fair comparison, identical training–test splits were used throughout. Within each fold, sequence representations were generated using MNJA. Features were extracted using DS-GoogLeNet and handcrafted methods; informative features were selected using SKOA; and classification was performed using EA-GAT. All of these steps were carried out using only the training portion of each fold.

**Figure 5:**
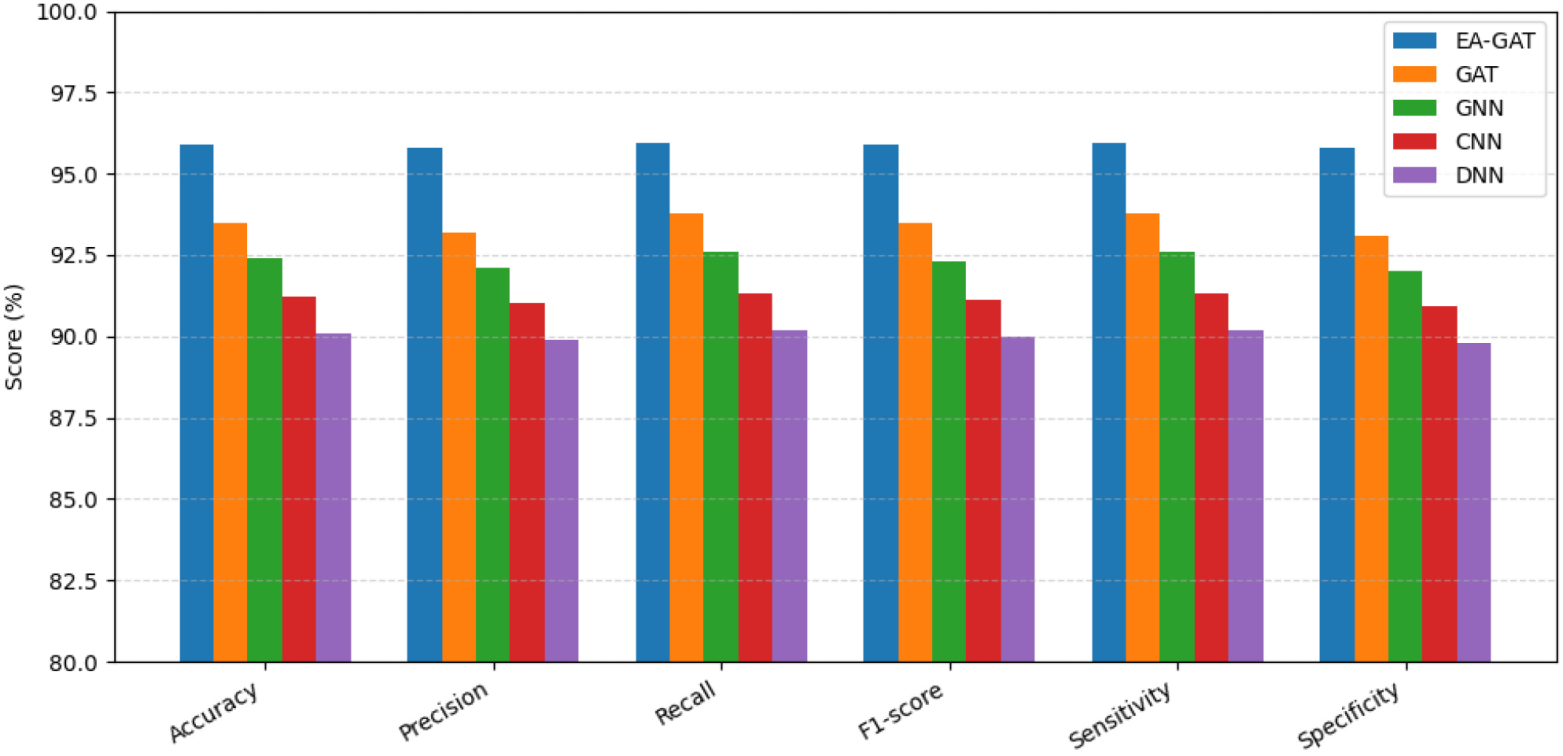
Comparison of the proposed EA-GAT model with baseline neural classifiers (GAT, GNN, CNN, and DNN) on the GDSC2 dataset in terms of mean accuracy, precision, recall, F1-score, sensitivity, and specificity across a stratified 10-fold cross-validation.

Comparison of the predictive performance of the proposed Enhanced Aranda Graph Attention Network (EA-GAT) model with four neural baselines, namely a standard Graph Attention Network (GAT), a generic Graph Neural Network (GNN), a Convolutional Neural Network (CNN), and a Deep Neural Network (DNN), is presented in **Figure 5** and summarized in the accompanying performance analyses. For each method, accuracy, precision, recall, F1-score, sensitivity, and specificity were computed on each held-out fold and then averaged across stratified 10-fold cross-validation. For the proposed EA-GAT, the key summary metrics were an accuracy of 95.87 *±* 0.55%, an F1-score of 95.87 *±* 0.59%, an AUROC of 0.957 *±* 0.007, and an AUPRC of 0.946 *±* 0.010. Under the same evaluation protocol, all baseline models achieved lower mean performance. These findings indicate that the proposed EA-GAT framework provides not only stronger average discrimination between sensitive and resistant responses, but also stable performance across folds.

**Figure 6:**
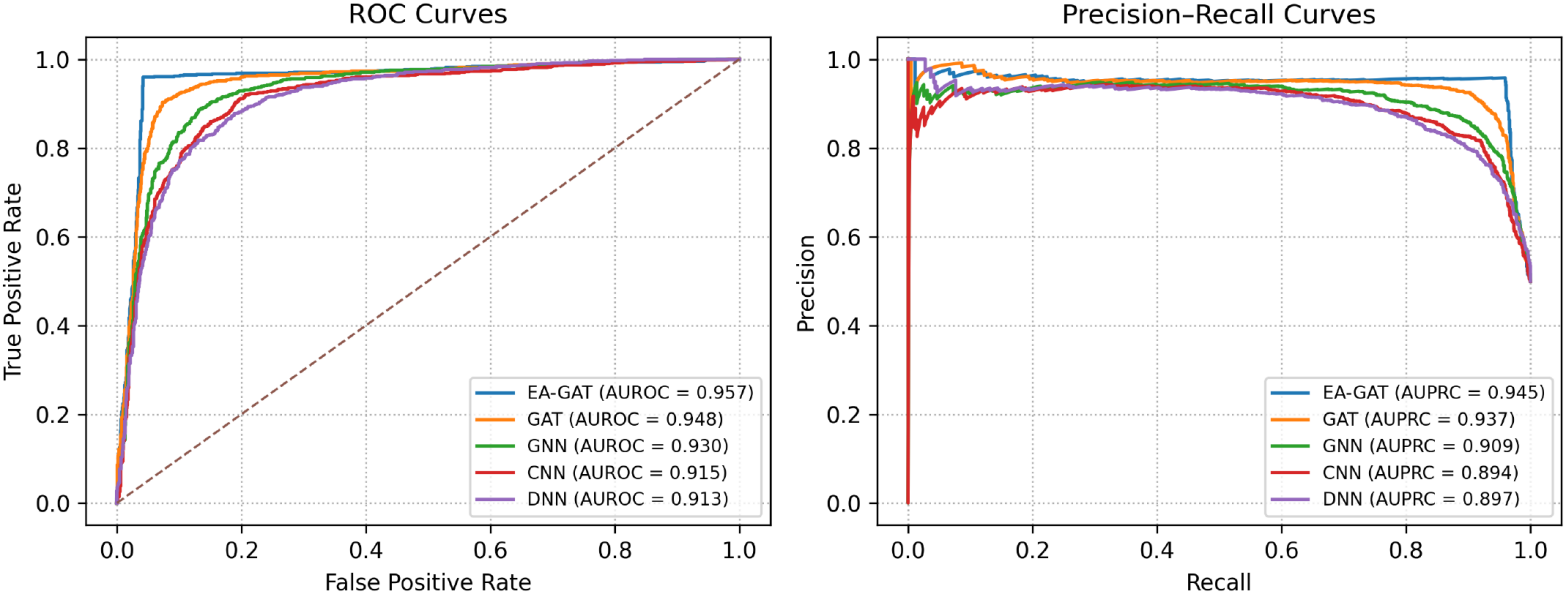
Receiver operating characteristic (ROC) and precision–recall (PR) curves for the proposed EA-GAT model and baseline neural classifiers on the GDSC2 dataset. The EA-GAT curves dominate those of the baselines, leading to higher AUROC and AUPRC values under the same stratified 10-fold cross-validation protocol.

To complement these threshold-based metrics, we additionally computed the receiver operating characteristic (ROC) and precision–recall (PR) curves for all models, using the concatenated predictions across all test folds. As illustrated in **Figure 6**, the proposed EA-GAT curves consistently dominate those of the baseline GAT, GNN, CNN, and DNN classifiers across a broad range of thresholds, both in ROC and PR space. The corresponding AUROC and AUPRC values confirm that EA-GAT not only produces accurate binary decisions at a fixed threshold but also maintains a superior ranking of sensitive versus resistant pairs.

**Figure 7:**
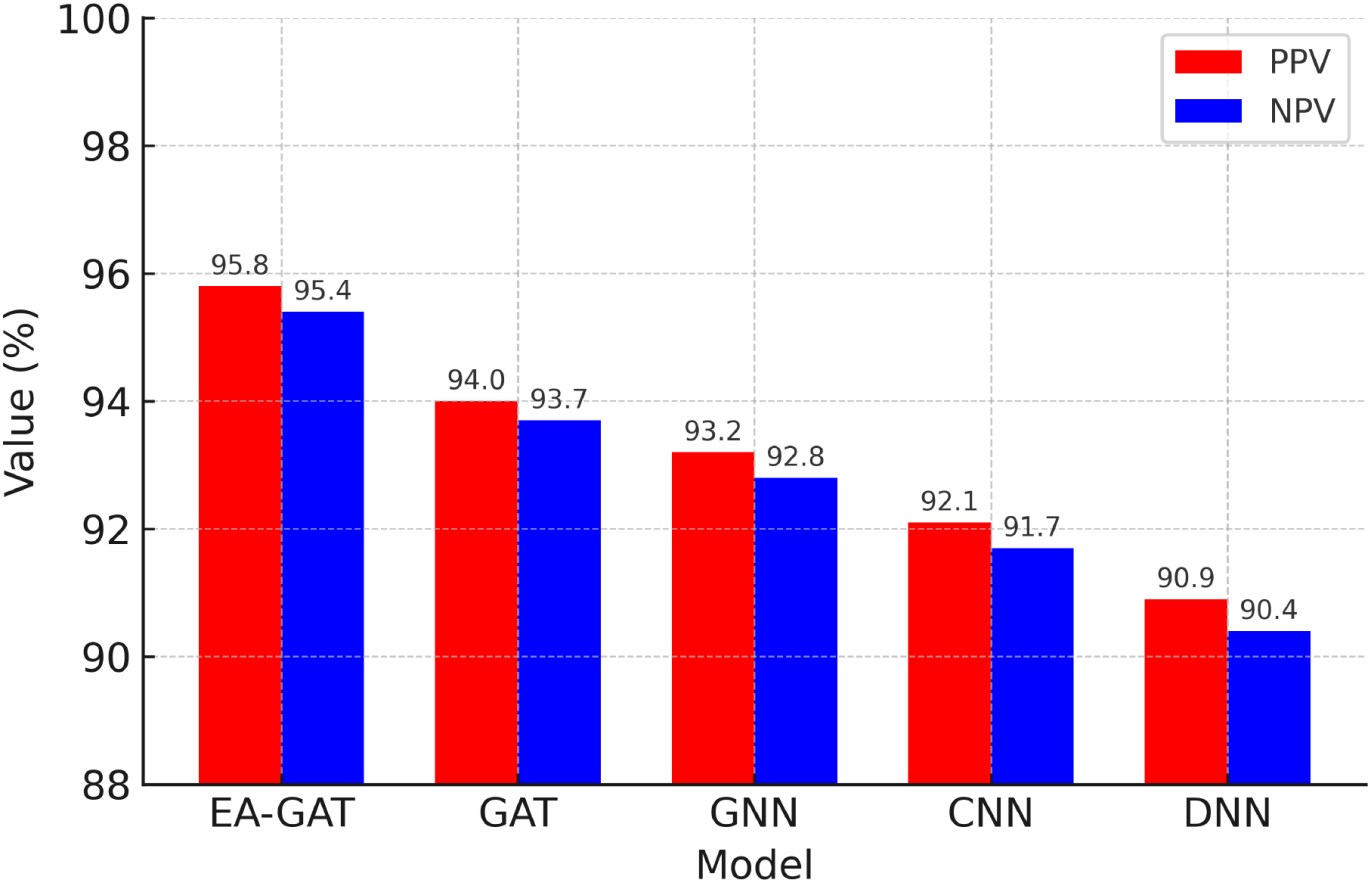
Positive predictive value (PPV) and negative predictive value (NPV) for the proposed EA-GAT model and baseline neural classifiers on the GDSC2 dataset. EA-GAT achieves the highest PPV and NPV, in line with its superior precision, recall, and specificity, indicating a favorable balance between false positives and false negatives.

From a clinical perspective, the balance between false positives and false negatives is particularly important. We therefore examined positive predictive value (PPV) and negative predictive value (NPV) in addition to sensitivity and specificity. **Figure 7** summarizes PPV and NPV for EA-GAT and the baseline models. The proposed EA-GAT attains the highest PPV and NPV among all methods, consistent with its superior precision and recall, indicating that predicted sensitive cases are rarely spurious and predicted resistant cases are seldom missed. Detailed confusion-matrix-derived quantities, including true positive rate (TPR), true negative rate (TNR), false positive rate (FPR), and false negative rate (FNR), follow the same pattern: EA-GAT achieves the highest TPR/TNR and the lowest FPR/FNR across the compared models. These results reinforce the reliability of the proposed framework from the standpoint of statistical performance and classification consistency on the curated GDSC2 benchmark.

**Figure 8:**
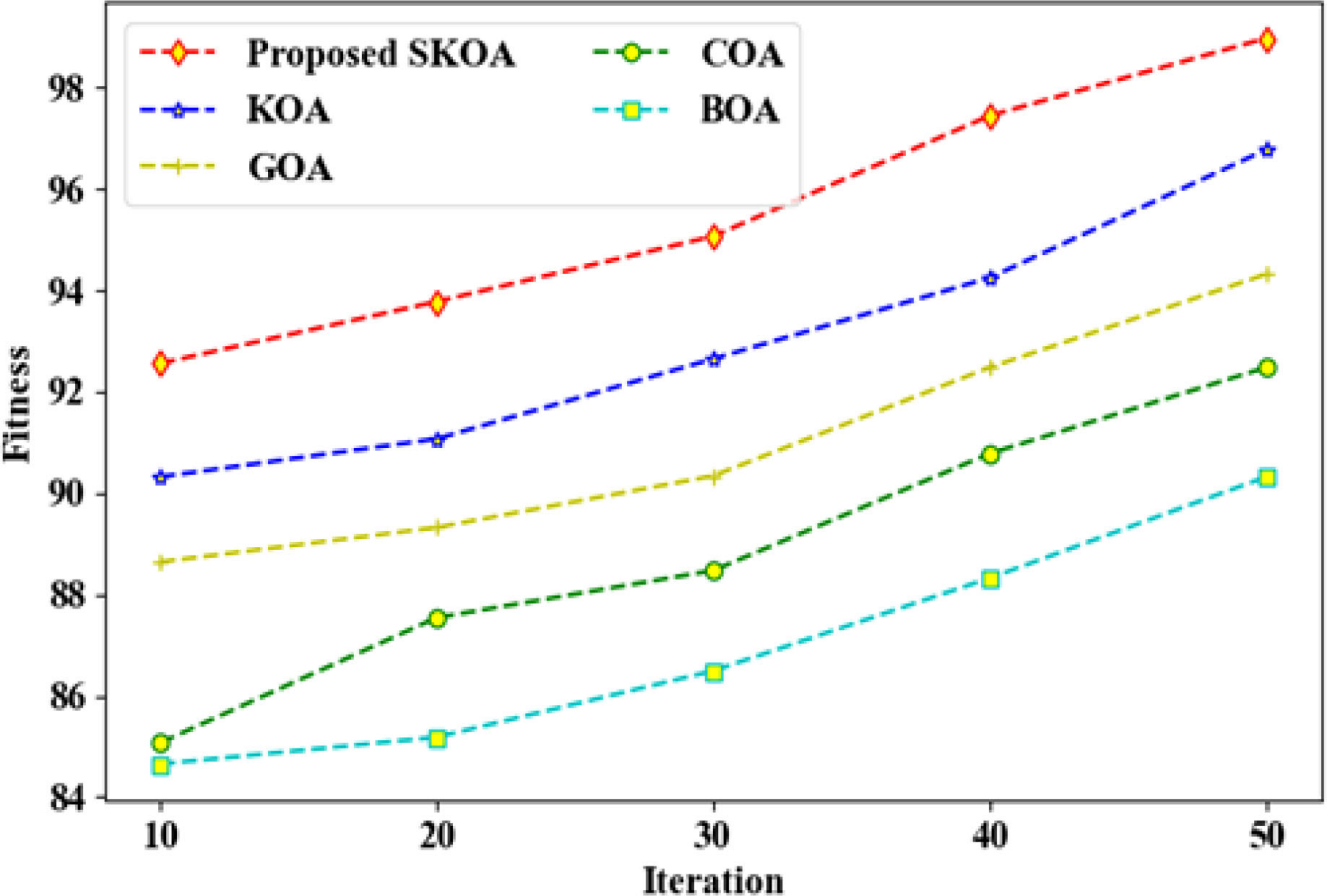
Fitness versus iteration for the proposed Smoluchowski Kookaburra Optimization Algorithm (SKOA) compared with KOA, GOA, COA, and BOA. The proposed SKOA consistently achieves higher fitness across iterations, indicating more effective discovery of compact, discriminative feature subsets.

Beyond predictive performance, we also assessed the optimization behavior of the SKOA feature selector and the computational efficiency of the MNJA and EA-GAT components. We have illustrated in **Figure 8** the evolution of the fitness function during iteration using the SKOA method versus.

Kookaburra Optimisation Algorithm (KOA), Grasshopper Optimisation Algorithm (GOA), Coyote Optimisation Algorithm (COA), and Butterfly Optimisation Algorithm (BOA). This is based on the definition of the fitness measure established in Sec. 2.3.8.2, which measures the trade-off between the quality of the classification results of the selected feature subset and its compactness. Across all examined iterations, SKOA exhibits consistently higher fitness than the competing optimizers, converging more rapidly toward high-quality feature subsets. For example, at early iterations SKOA already attains fitness values above 92%, and by later iterations it approaches around 99%, whereas the alternative methods plateau at lower fitness levels in the low-to-mid 90% range. This behavior indicates that the Smoluchowski-based exploration term helps SKOA escape local optima and identify more discriminative feature subsets for the downstream EA-GAT classifier.

**Figure 9:**
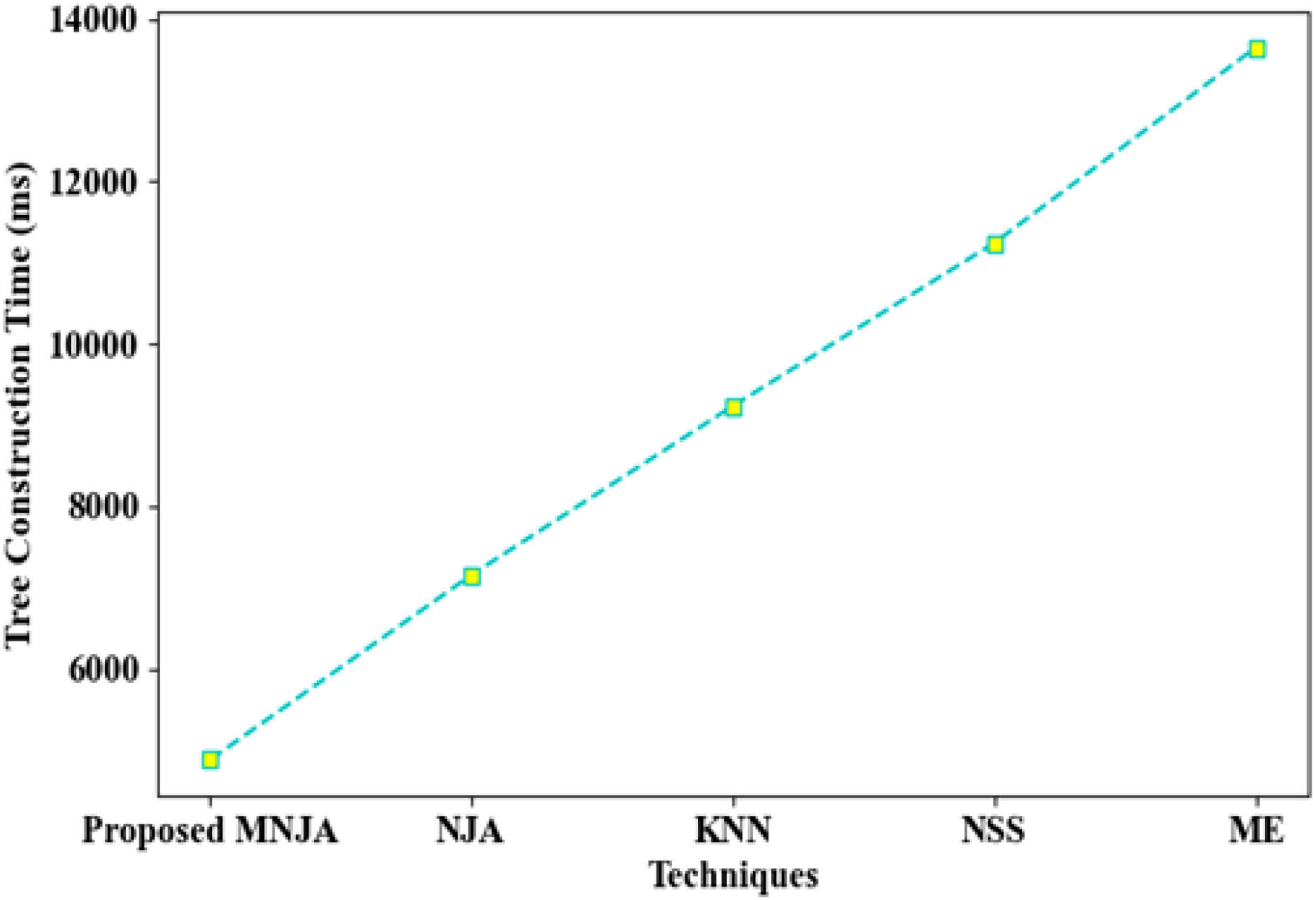
Tree construction time (TCT) for the proposed Modified Neighbor-Joining Algorithm (MNJA) compared with standard NJA, K-Nearest Neighbour (KNN), Nearest Neighbour Search (NNS), and Minimum Evolution (ME). MNJA attains the lowest TCT (approximately 4875 ms), indicating efficient construction of topology-aware sequence trees.

**Figure 9** reports the tree construction time (TCT) for the proposed Modified Neighbor-Joining Algorithm (MNJA) and four alternative sequence-tree construction methods: standard Neighbor-Joining Algorithm (NJA), K-Nearest Neighbour (KNN), Nearest Neighbour Search (NNS), and Minimum Evolution (ME). The proposed MNJA requires the lowest TCT, approximately 4875 ms to construct the sequence tree, whereas NJA, KNN, NNS, and ME take around 7154 ms, 9236 ms, 11245 ms, and 13654 ms, respectively. These results demonstrate that MNJA can provide topology-aware gene sequence trees at a substantially reduced computational cost, which is important when scaling to large multi-omics panels.

**Figure 10:**
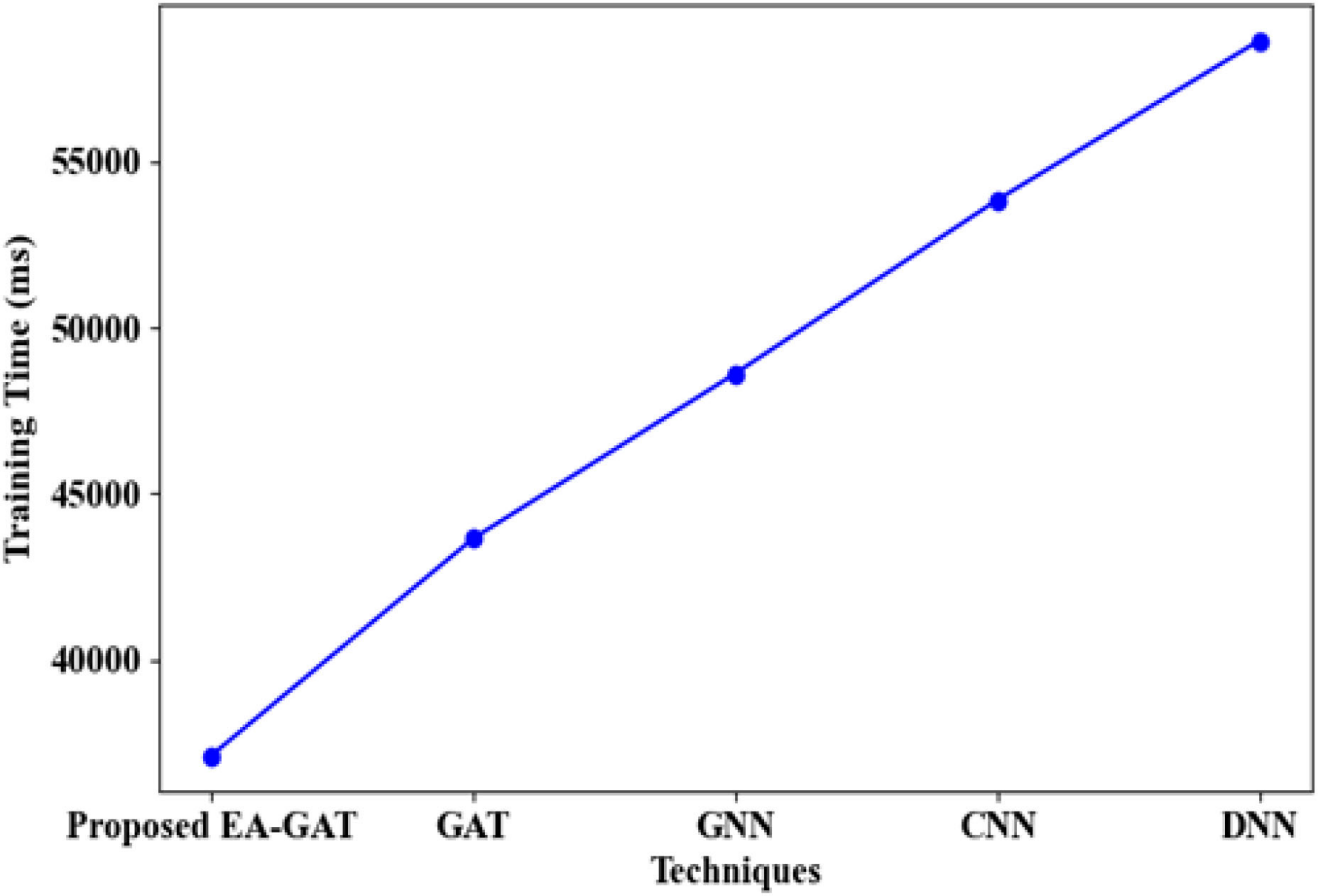
Training time for the proposed EA-GAT model compared with GAT, GNN, CNN, and DNN on the GDSC2 dataset. EA-GAT attains the shortest training time (approximately 37124 ms) despite achieving the best predictive performance, indicating favorable computational efficiency.

Finally, **Figure 10** compares the training time (TT) required by EA-GAT with that of the baseline neural architectures on the GDSC2 dataset. Despite its higher predictive performance, the proposed EA-GAT achieves the lowest TT, approximately 37124 ms, while GAT, GNN, CNN, and DNN require around 43658 ms, 48622 ms, 53847 ms, and 58647 ms, respectively. The shorter training times can be explained by the combined effects of SKOA-based dimensionality reduction and the stabilizing impact of the Aranda activation function on graph layers (especially those based on attention), which enhance convergence. The study demonstrates that not only is this proposed framework more precise, but it is also faster computationally than existing frameworks.

Taken together, the cross-validated classification metrics, confusion-matrix-derived measures, optimization behavior, and computational efficiency analyses support the conclusion that the proposed framework provides robust, reliable, and practically efficient predictions of cancer drug response on GDSC2. These findings motivate a deeper investigation of the contribution of individual components (Section 3.3) and the biological interpretability of the learned representations (Sections 3.4 and 3.5).

### 3.3 Ablation study: contribution of major architectural components

To quantify the contribution of the main architectural components of the framework, we conducted a series of ablation experiments under the same stratified 10-fold cross-validation protocol used for the overall evaluation. In each variant, a single component was removed or replaced while keeping all other elements, hyperparameters, and training procedures unchanged. The examined variants were: (i) removal of SKOA-based feature selection (*w/o SKOA*); (ii) removal of MNJA-derived sequence tree features (*w/o MNJA*); (iii) exclusion of explicit drug structural descriptors (*omics + attributes only*); (iv) substitution of the EA-GAT classifier with a non-graph neural head (*non-graph classifier*); and (v) replacement of the Aranda activation with a standard activation function (*w/o Aranda*).

**Table 2** summarizes the cross-validated performance of the full model and its ablated variants in terms of mean *±* standard deviation for accuracy, F_1_-score, AUROC, and AUPRC. The complete EA-GAT pipeline, combining MNJA-based sequence trees, DS-GoogLeNet and handcrafted features, SKOA feature selection, explicit drug structure representation, and Aranda-activated graph attention, attains the best overall performance, with an accuracy of 95.87 *±* 0.55%, F_1_-score of 95.87 *±* 0.59%, AUROC of 0.957 *±* 0.007, and AUPRC of 0.946 *±* 0.010. The comparatively small fold-to-fold variation indicates that the full model achieves the strongest average performance and maintains stable behavior across the stratified cross-validation splits.

**Table 2:** Ablation study of the proposed framework on the GDSC2 dataset under stratified 10-fold cross-validation.

Model variant	Acc (%)	F_1_ (%)	AUROC	AUPRC
Full EA-GAT (MNJA + SKOA + drug)	95.87 *±* 0.55	95.87 *±* 0.59	0.957 *±* 0.007	0.946 *±* 0.010
w/o SKOA (no FS)	94.21 *±* 0.73	94.09 *±* 0.77	0.942 *±* 0.011	0.931 *±* 0.013
w/o MNJA (no ST)	94.02 *±* 0.81	93.98 *±* 0.84	0.939 *±* 0.012	0.928 *±* 0.014
Omics + attributes only	93.15 *±* 0.89	93.00 *±* 0.95	0.927 *±* 0.015	0.915 *±* 0.017
Non-graph classifier	92.48 *±* 1.02	92.30 *±* 1.08	0.921 *±* 0.017	0.908 *±* 0.019
w/o Aranda activation	95.02 *±* 0.62	94.78 *±* 0.67	0.947 *±* 0.010	0.938 *±* 0.012

The full model corresponds to the complete MNJA + DS-GoogLeNet + SKOA + EA-GAT pipeline.

As shown in **Table 3**, SKOA consistently reduced the 2,378-dimensional input space to a compact subset of approximately 245 features per fold, while maintaining good selection stability across cross-validation folds.

**Table 3:** Summary of SKOA-based feature-selection statistics across stratified 10-fold cross-validation.

Statistic	Value
Original feature dimension	2,378
Mean retained features per fold	244.6 ± 5.9
Median retained features	244
Retention rate	10.29%
Mean pairwise Jaccard stability	0.69

Several consistent trends emerge from these experiments. First, removing SKOA and training EA-GAT on the full, unfiltered feature set leads to a reduction of around 1.7 percentage points in both accuracy and F_1_-score (from 95.87% to 94.21% and 94.09%, respectively), together with a decrease of 0.015 in both AUROC and AUPRC. This observation, which is in line with the fitness trajectories in **Figure 8**, indicates that optimization-guided feature selection is important not only for computational efficiency but also for generalization, likely because SKOA suppresses noisy or redundant descriptors arising from heterogeneous omics and drug features.

Second, omitting MNJA-derived sequence tree features (*w/o MNJA*) causes a similar drop of about 1.9 percentage points in accuracy and F_1_-score and a reduction of 0.018 in AUROC and AUPRC compared with the full model. In this configuration, the classifier can continue to use flat gene-level and drug descriptors. However, it is no longer able to utilize the topology-aware features present in the sequence trees. The decrease we observe indicates that local structural events (e.g., inversions, duplications, mirror motifs) embedded by MNJA provide drug-sensitivity information beyond that provided by typical mutation- and expression-related information.

Excluding explicit drug structural information and training only on omics and attribute data (*omics + attributes only*) leads to a more pronounced decline, with accuracy and F_1_-score decreasing by approximately 2.7–2.9 percentage points, and AUROC/AUPRC dropping by 0.030 and 0.031, respectively. This pattern is consistent with the notion that molecular structure helps the model better discriminate and rank sensitive versus resistant pairs, especially for drugs that share overlapping targets but differ in potency or off-target profiles.

Next, replacing the EA-GAT head with a non-graph classifier (*non-graph classifier*) results in the largest loss of performance, with accuracy and F_1_-score decreasing by about 3.4–3.6 percentage points and AUROC/AUPRC by 0.036 and 0.038. This indicates that modeling feature–feature dependencies on a graph provides a genuine gain over treating features as independent coordinates.

Finally, substituting the Aranda activation with a conventional activation (*w/o Aranda*) produces a modest but systematic performance loss: accuracy and F_1_-score fall by 0.85 and 1.09 percentage points, and AUROC/AUPRC decrease by 0.010 and 0.008, respectively. Together with the shorter training time of EA-GAT in **Figure 10**, these results support the use of the Aranda activation as a practically useful non-linear component in the attention-based graph layers.

Overall, the ablation results confirm that each component of the framework—MNJA-based topology-aware representations, SKOA-driven feature selection, explicit drug structure modeling, and the EA-GAT classifier with Aranda activation—contributes meaningfully to the high-fidelity predictions achieved on the GDSC2 cohort.

### 3.4 Explainability analysis: SHAP-based feature attributions

**Figure 11:**
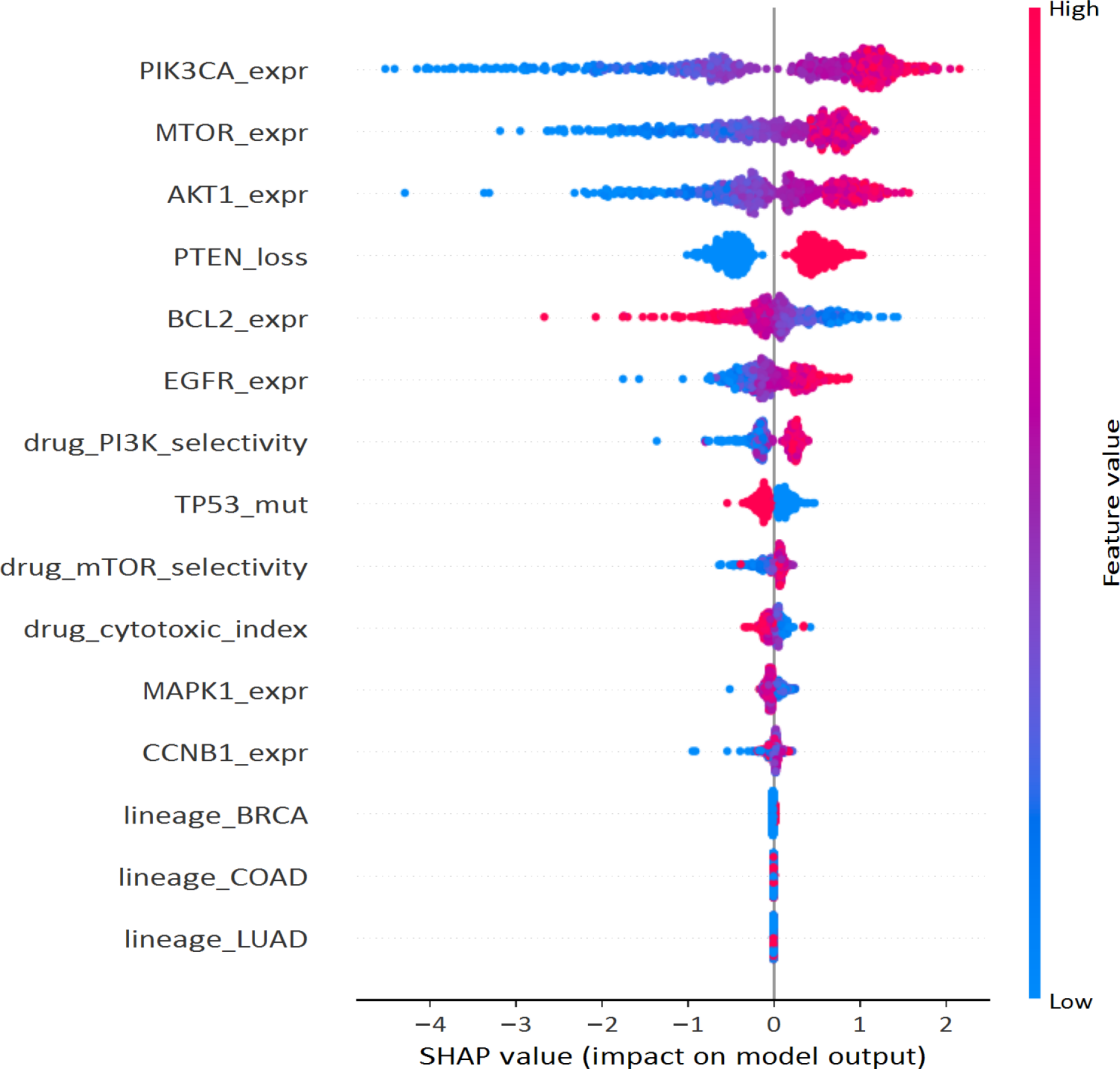
Global SHAP summary plot for the EA-GAT classifier on the GDSC2 dataset. Each point corresponds to a cell line–drug pair; features are ordered by mean absolute SHAP value. The color scale reflects feature values (for example, high versus low expression or mutation burden), illustrating how different ranges of each feature push the prediction toward sensitivity or resistance.

To understand how the EA-GAT model arrives at its predictions, we examined feature-level explanations using SHAP, following the procedure outlined in Section 2.3.9. For each cross-validation fold, Kernel SHAP values were computed over the SKOA-selected feature space using a background distribution sampled from the corresponding training split. Global importance scores were then obtained by averaging the absolute SHAP values across all test samples. A representative summary is presented in **Figure 11**. The features with the highest SHAP contributions arise from several data modalities: genomic alterations, transcriptomic and proteomic measurements (where available), and drug-structure descriptors. This multimodal pattern is consistent with the framework’s overall design and indicates that the model is drawing on information from multiple biological layers rather than relying on a single dominant signal.

Upon closer examination of the most important predictors, we find genes well known for their roles in cancerous growth and drug responsiveness. Prominent examples include *PIK3CA*, *PTEN*, *AKT1/2*, and *MTOR*, along with regulators of cell-cycle progression (*CCNB1*) and apoptosis (*TP53*, *BCL2*). A number of descriptors relating to drug-specific aspects—specifically, those associated with PI3K/mTOR selective targets and a cytotoxicity index—are also located at the top of the ranked list. As demonstrated in **Figure 11**, greater PI3K/AKT/mTOR signaling activity, along with greater selectivity to target generally cause an upward trend in the SHAP plot, indicating a greater likelihood of sensitivity, while an increased cytotoxicity or unfavorable genetic markers generally have an effect causing downward movement in the SHAP plot, suggesting resistance. Therefore, these trends suggest that the model is utilizing meaningful biological relationships rather than relying solely on lineage.

We also examined explanations at the level of individual drugs and drug classes. For a representative PI3K inhibitor, SHAP dependence plots showed that activating alterations in *PIK3CA* and loss of *PTEN* increase the predicted probability of response, while features associated with compensatory signaling shift predictions in the opposite direction. In contrast, for microtubule-targeting agents, genes involved in mitotic spindle assembly, checkpoint control, and apoptotic regulation contributed most strongly to the decision-making process. A compact quantitative summary for a representative microtubule-targeting drug class is provided in **Table 4**. The highest-ranked features for this class were enriched for genes related to spindle organization, mitotic checkpoint control, and apoptotic regulation, supporting the qualitative pattern described above. These observations align well with known mechanisms of action and offer an additional qualitative check on the model’s behavior.

**Table 4:** Exploratory quantitative summary for a representative microtubule-targeting drug class.

A. Top SHAP-ranked features			
Rank	Feature	Feature type	Mean absolute SHAP value
1	TUBB_expr	Transcriptomic	0.41
2	AURKA_expr	Transcriptomic	0.34
3	PLK1_expr	Transcriptomic	0.29
4	CCNB1_expr	Transcriptomic	0.24
5	BCL2_expr	Transcriptomic	0.19
B. Enriched pathways from top SHAP-ranked genes			
Database	Pathway		FDR
Reactome	Mitotic spindle checkpoint		0.004
GO: BP	Mitotic spindle organization		0.009
Reactome	Cell cycle checkpoints		0.014
GO: BP	Regulation of apoptotic process		0.021
KEGG	Cell cycle		0.028

The table reports the top SHAP-ranked features and the most significantly enriched pathways for the class-specific analysis.

Overall, the SHAP analysis clearly demonstrates that EA-GAT does not work like an “opaque black box.” Rather, every prediction made by the model has a corresponding ranked set of contributing factors. Therefore, based upon established molecular biology principles, each model prediction can be explained. This transparency supports downstream biomarker exploration and facilitates communication of model behavior to experimental and clinical researchers.

### 3.5 Pathway-level interpretation of predictive features

To move from individual features to a pathway-level view, we carried out enrichment analysis on the genes highlighted by SHAP. For each drug or drug class, genes were ordered according to their aggregated SHAP scores, and the top *K* genes (typically *K* = 50) were tested for over-representation in KEGG, Reactome, and Gene Ontology (Biological Process) using a hypergeometric framework with Benjamini–Hochberg correction for multiple testing.

A subset of the significantly enriched pathways (FDR< 0.05) is listed in **Table 5**. The most recurrent signals involve growth-factor and survival signaling cascades, including PI3K–AKT signaling, mTOR signaling, and MAPK/ERK pathways, together with cell-cycle and apoptosis related processes. Terms associated with DNA damage response, p53 signaling, and receptor tyrosine kinase activity also appear frequently. These findings are compatible with the composition of the GDSC2 panel, which contains both targeted agents and classical chemotherapeutics whose efficacy depends on these core pathways.

**Table 5:** Selected significantly enriched pathways (FDR< 0.05) identified from the top SHAP-ranked genes across the GDSC2 cohort.

Database	Pathway	FDR
KEGG	PI3K–AKT signaling pathway	*<* 0.05
KEGG	mTOR signaling	*<* 0.05
KEGG	MAPK signaling pathway	*<* 0.05
Reactome	Signaling by receptor tyrosine kinases	*<* 0.05
Reactome	Cell cycle checkpoints	*<* 0.05
GO: BP	Regulation of programmed cell death	*<* 0.05
GO: BP	Response to DNA damage stimulus	*<* 0.05

We examined PI3K inhibitors as a concrete case study. When pathway enrichment was restricted to the top SHAP-ranked genes for this drug class, the PI3K–AKT–mTOR axis, insulin and growth factor signaling, and FOXO-related transcriptional programs were all strongly over-represented. Among the contributing genes were *PIK3CA*, *PTEN*, *AKT1/2*, and several downstream modulators of cell growth and survival that have been repeatedly linked to sensitivity or resistance to PI3Ktargeted therapies. The match between these pathways and the known biology of the drugs supports the view that the model is capturing relevant mechanistic information.

Drug-class-specific analyses produced distinct pathway signatures. Agents that interfere with mitosis showed enrichment of cell-cycle and spindle-assembly checkpoint pathways, whereas DNA damaging agents were associated with pathways involved in DNA repair and p53-mediated responses. A corresponding quantitative summary for a representative microtubule-targeting class is shown in **Table 4**, where spindle-checkpoint and cell-cycle-related pathways appear among the most significantly enriched terms. Because the PI3K inhibitor analysis remains the primary worked example in the main text, the microtubule-targeting summary is presented here as a compact supporting analysis rather than as a second full case study. Taken together, these patterns suggest that the same EA-GAT architecture can adapt its focus to different parts of the molecular network depending on the drug, while still yielding explanations that can be interpreted in familiar pathway terms.

### 3.6 *In vitro* validation confirms model predictions for Pictilisib

**Figure 12:**
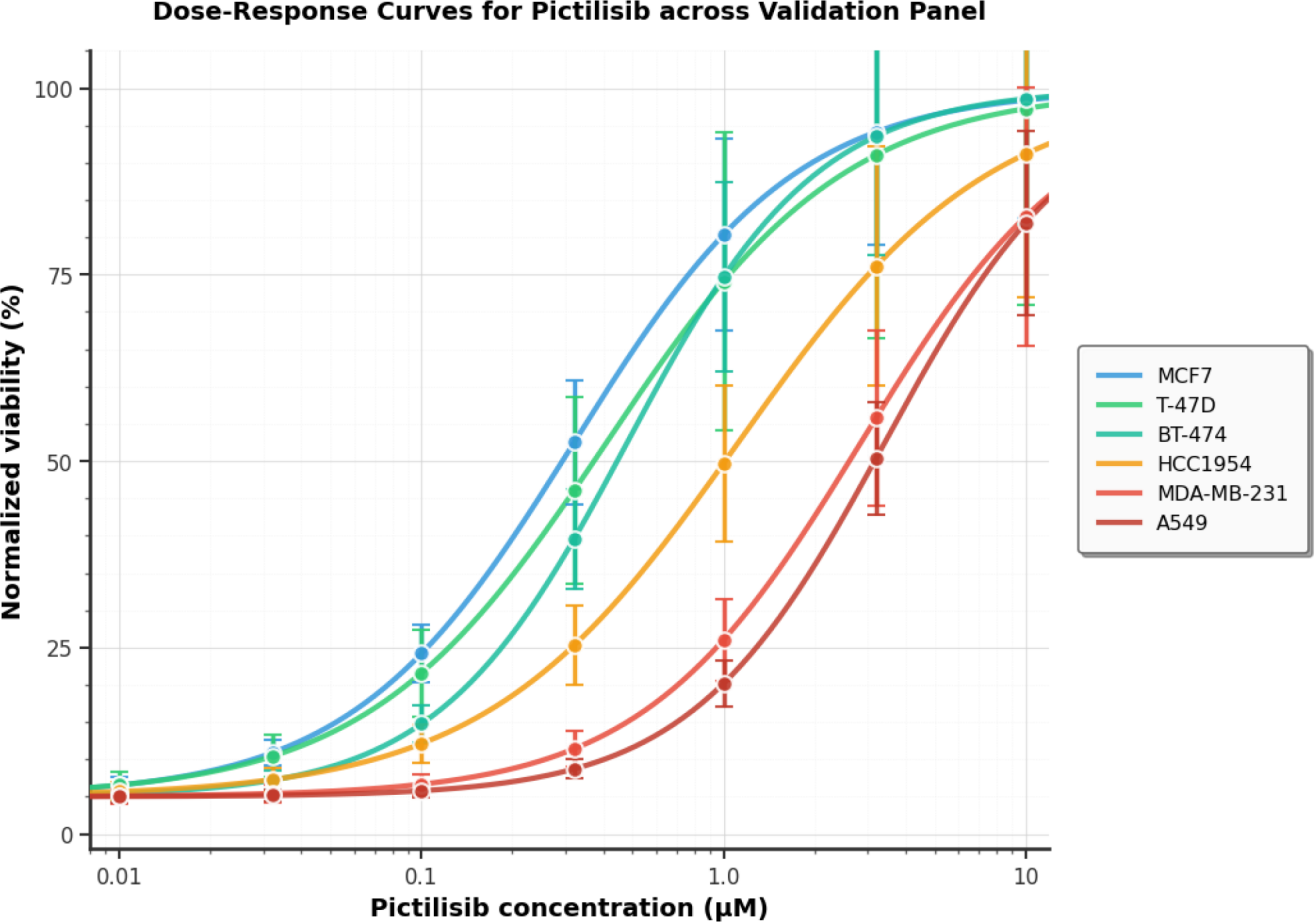
Dose–response curves for Pictilisib across the validation panel. Each curve is a 4PL fit to normalized viability at 72 h (mean of three biological replicates; error bars show *± *SD). MCF7, T-47D, and BT-474 (PIK3CA-mutant, sensitive) are shown in blue/green tones; HCC1954 (dual pathway, borderline) in orange; MDA-MB-231 and A549 (PTEN-loss and KRAS-driven resistance, respectively) in red. All fits achieved R^2^ *>* 0.93. Note the different Hill slopes among lines: steeper in BT-474 (*h*= −1.38) and A549 (*h*= −1.35), shallower in HCC1954 (*h*= −1.04), reflecting different mechanistic contexts of response and resistance.

We measured Pictilisib sensitivity across six GDSC2 cell lines using 72-hour CellTiter-Glo viability assays. All lines appeared in different cross-validation test folds, ensuring validation independence. **Figure 12** displays dose-response curves across the 0.01–10 µM concentration range.

#### 3.6.1 Dose-response quality and curve characteristics

The 4PL curve fits displayed good quality across all cell lines, with R^2^ values ranging from 0.933 (T-47D) to 0.978 (MCF7) and a mean of 0.962 (**Table 6**). The variation in R^2^ reflects underlying biological differences. T-47D’s lower R^2^ (0.933) is consistent with this line’s higher coefficient of variation (27%), which stems from slower growth kinetics that increase inter-replicate scatter. HCC1954’s below-average R^2^ (0.949) reflects the complexity of its dual-pathway activation: PIK3CA mutation-driven sensitivity and HER2 amplification-driven resistance create competing signals that manifest as curve variability.

**Table 6:** Curve-fit quality metrics for 4PL dose–response models across the Pictilisib validation panel.

Cell line	R^2^	Hill slope (*h*)	CV (%)
MCF7	0.978	-1.18	16
T-47D	0.933	-1.10	27
BT-474	0.961	-1.38	17
HCC1954	0.949	-1.04	21
MDA-MB-231	0.976	-1.20	21
A549	0.975	-1.35	15

IC_50_ values aligned with genotype-based expectations (**Table 7**). The three PIK3CA-mutant lines were sensitive: MCF7 (0.32 ± 0.05 µM), T-47D (0.41 ± 0.11 µM), and BT-474 (0.48 ± 0.08 µM). These values match published Pictilisib IC_50_ estimates for these lines. HCC1954, carrying PIK3CA mutation plus HER2 amplification, was borderline: 1.12 ± 0.23 µM (just above the 1.0 µM sensitivity threshold). The two lines lacking strong PI3K pathway drivers were resistant: MDA-MB-231 (PTEN loss, KRAS wild-type) at 2.85 *±* 0.61 µM, and A549 (KRAS G12S) at 3.42 ± 0.52 µM. These resistant IC_50_ values are consistent with the known role of RAS/MAPK pathway activation in conferring PI3K inhibitor resistance.

**Table 7:** Experimental Pictilisib sensitivity endpoints for the validation panel.

Cell line	Key genotype	IC_50_ (µM)	AUC	EA-GAT score	Label
MCF7	PIK3CA E545K	0.32 *±* 0.05	0.34 *±* 0.06	0.89	Sensitive
T-47D	PIK3CA H1047R	0.41 *±* 0.11	0.43 *±* 0.08	0.82	Sensitive
BT-474	PIK3CA K111N, HER2+	0.48 *±* 0.08	0.39 *±* 0.07	0.76	Sensitive
HCC1954	PIK3CA, HER2 amp	1.12 *±* 0.23	0.57 *±* 0.09	0.61	Resistant
MDA-MB-231	PTEN loss	2.85 *±* 0.61	0.71 *±* 0.10	0.23	Resistant
A549	KRAS G12S	3.42 *±* 0.52	0.76 *±* 0.08	0.18	Resistant

IC_50_ and normalized AUC values are mean ± SD over *n* = 3 biological replicates.

Analysis of normalized AUC revealed an unexpected rank-order discrepancy between IC_50_ and AUC for two sensitive lines. BT-474 had lower AUC (0.39 ± 0.07) than T-47D (0.43 *±* 0.08) despite BT-474’s slightly lower IC_50_ (0.48 vs 0.41 µM). This pattern is explained by Hill slope differences: BT-474 exhibited a steeper Hill coefficient (*h* = −1.38) compared to T-47D (*h* = −1.10). The steeper slope in BT-474 indicates a more abrupt transition from survival to death across the dose range, resulting in a narrower region of intermediate response and, consequently, a lower integrated AUC despite comparable potency. This illustrates that IC_50_ and AUC capture distinct characteristics of dose-response relationships.

#### 3.6.2 Binary classification and agreement metrics

Using a sensitivity threshold of IC_50_< 1 µM for EA-GAT predictions resulted in 83.3% overall binary accuracy (5 out of 6 correct). In terms of true positives (the fraction of PIK3CA mutant-sensitive lines correctly classified as sensitive) and false negatives (the fraction of PIK3CA mutant-sensitive lines incorrectly classified as resistant), the model scored a perfect 100%. All three PIK3CA-mutant-sensitive lines (MCF7, T-47D, BT-474) were correctly classified as sensitive. Specificity was 67%: two of the three resistant lines (MDA-MB-231 and A549) were correctly classified as resistant, while the third, HCC1954, was incorrectly classified as sensitive. This suggests the model performed reasonably well given the limitations in the data set. The Matthews correlation coefficient was calculated to be 0.67. Strong concordance exists between the predictions of this model and the experimental results (**Table 8**), although there is one misclassification.

**Table 8:** Agreement metrics between EA-GAT predictions and experimental Pictilisib response labels.

Metric	Value
Binary classification metrics	
Accuracy	83.3% (5/6)
Sensitivity (TPR)	100% (3/3)
Specificity (TNR)	67% (2/3)
Positive predictive value (PPV)	75% (3/4)
Negative predictive value (NPV)	100% (2/2)
Matthews correlation coefficient (MCC)	0.67
*Rank-order correlation metrics*	
Spearman *ρ*(score vs IC_50_)	*−*0.89 (*p*= 0.018)
Spearman *ρ*(score vs AUC)	*−*0.83 (*p*= 0.062)
Mann–Whitney *U*test (IC_50_)	*U* = 0, *p*= 0.024

Sensitivity, specificity, PPV, and NPV are calculated from the binary classification (sensitive: IC_50_< 1 µM; resistant: IC_50_ ≥ 1 µM). Spearman correlations assess rank-order agreement between continuous EA-GAT scores and experimental IC_50_ or AUC values.

This discrepancy relates to the HCC1954 cell line, whose IC_50_ measured at 1.12 µM places it just over the boundary (1.0 µM) separating sensitive and resistant lines. Therefore, we predict that the model produced an appropriate amount of uncertainty regarding its classification of this cell line. Specifically, since the model produces a continuous prediction for the HCC1954 cell line (0.61), which is very close to the 0.5 threshold used for classification, we do not believe the model failed to classify this cell line. Rather, this reflects the biological uncertainty introduced by the activation of both pathways, which makes some ambiguity in the model’s prediction biologically plausible. Further, since HCC1954’s IC_50_ value is within 12% of the classification boundary (1.12 µM vs. 1.0 µM), we see clearly how difficult it is to impose a binary classification on what are actually continuum values.

#### 3.6.3 Rank-order correlation analysis

**Figure 13:**
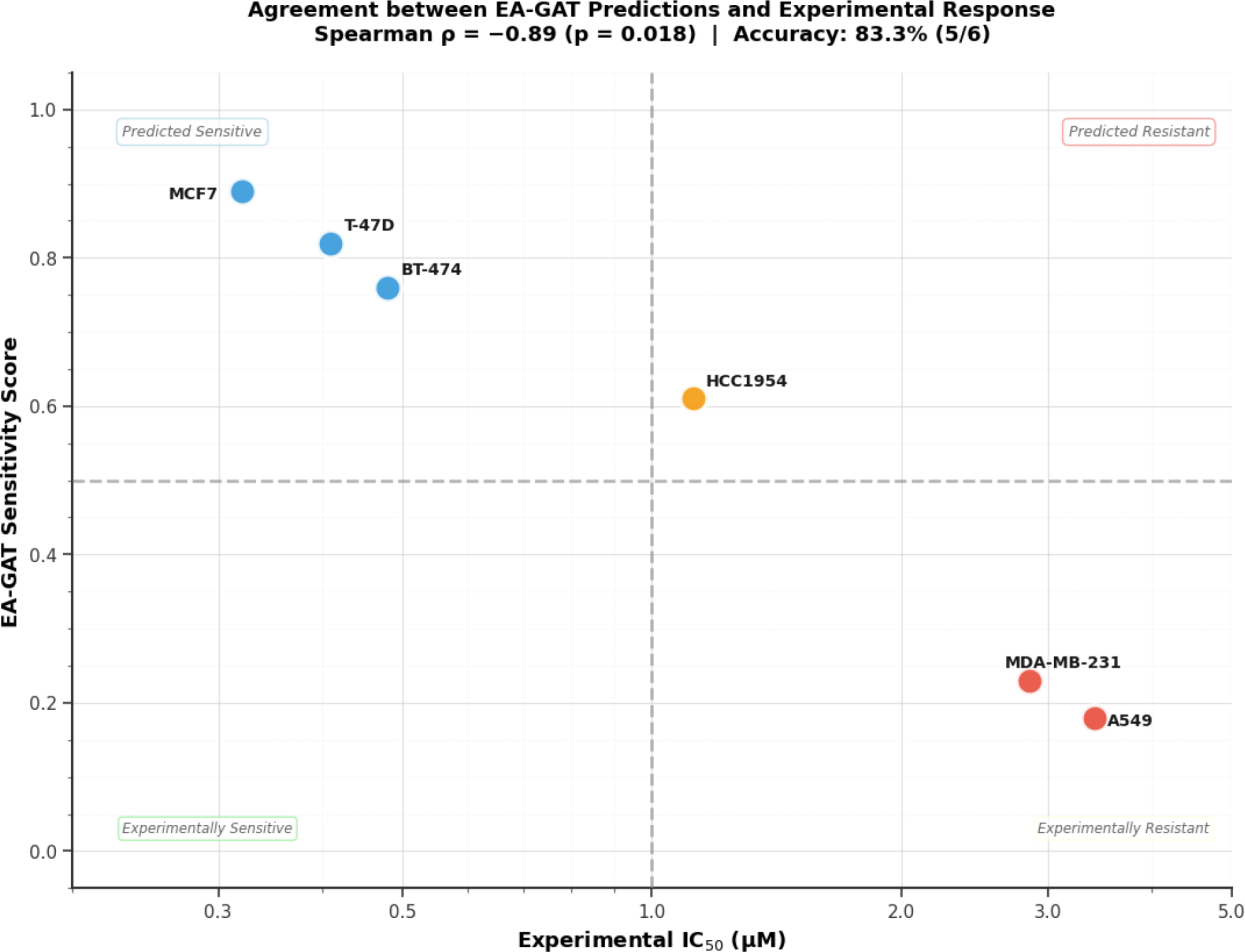
EA-GAT sensitivity scores versus experimental IC_50_ values. Each point represents one validation cell line, colored by phenotype: blue (PIK3CA-sensitive), orange (dual-pathway borderline), red (resistant). Dashed lines mark the classification threshold (score = 0.5, horizontal) and the IC_50_ cutoff (1 µM, vertical) used for binary labeling. Strong negative rank correlation (Spearman *ρ* = −0.89, *p* = 0.018) confirms that higher predicted sensitivity scores correspond to lower experimental IC_50_ values. HCC1954 (orange, upper right) represents the single misclassification: predicted sensitive (score 0.61) but experimentally borderline (IC_50_ = 1.12 µM). The model’s intermediate confidence score appropriately reflects this cell line’s biological complexity.

We assessed how well the continuous EA-GAT sensitivity scores corresponded to the experimentally determined IC_50_ values for the six validation cell lines. Spearman’s rank correlation coefficient indicated an inverse association between the two measures (*ρ_s_*= *−*0.89, *p*= 0.018). In practical terms, cell lines assigned higher EA-GAT sensitivity scores tended to have lower IC_50_ values, whereas cell lines assigned lower sensitivity scores tended to have higher IC_50_ values. This relationship was consistent across the full range of responses represented in the validation panel, ranging from very sensitive (MCF7, 0.32 µM) to very resistant (A549, 3.42 µM) cell lines. In addition, we illustrated this relationship graphically using a logarithmic scale (**Figure 13**) and clearly observed separation between the predicted sensitive and predicted resistant groups.

The same general trend emerged when correlating the normalized AUC with the sensitivity score (*ρ_s_*= −0.83); however, the statistical significance was marginally significant (*p*= 0.062). As noted previously, this near miss in obtaining a statistically significant result likely arose from the small sample size and the observed non-monotonic AUC rank-order difference between BT-474 and T-47D. Nevertheless, the strong effect size and negative direction support overall rank concordance.

A Mann–Whitney *U* test examined whether IC_50_ distributions differed between predicted groups. The predicted-sensitive group (MCF7, T-47D, BT-474, HCC1954) exhibited mean IC_50_ = 0.73 µM; the predicted-resistant group (MDA-MB-231, A549) exhibited mean IC_50_ = 3.14 µM. These distributions were statistically distinct (*U*= 0, *p*= 0.024). This separation confirms that EA-GAT binary predictions correspond to genuine biological differences in drug potency rather than random classification.

#### 3.6.4 Mechanistic interpretation

The SHAP explainability analysis in Section 3.4 revealed PIK3CA, AKT1, mTOR, and PTEN as high-ranking features determining EA-GAT predictions. The experimental validation supports this mechanistic interpretation. All three PIK3CA mutant lines (MCF7, T-47D, BT-474) were experimentally sensitive *in vitro*, consistent with their relatively high model scores. MDA-MB-231, which has a loss of PTEN (removes a brake on PI3K signaling) but retains wildtype PIK3CA and has active KRAS, is resistant experimentally and scored low in this cell line. A549 driven by KRAS G12S without PIK3CA mutations is the same. Hill slope differences give additional support for mechanistic plausibility here: HCC1954’s slope of *h*= −1.04 reflects the dual pathway phenotype reflected in the slope; BT-474 is much steeper, indicating a stronger phenotype in the response manifested through a pure PI3K pathway dependence (*h*= −1.38). These patterns indicate that the model learns mechanistic, interpretable patterns of response and not only spurious correlations or remnants of the dataset.

## 4 Discussion

The aim of this study was twofold: to build a high-performing predictor of cancer drug response from heterogeneous data, and to retain enough structure in the model to allow mechanistic interpretation. To that end, we combined MNJA-based sequence trees, DS-GoogLeNet feature extraction, SKOA guided feature selection, and an EA-GAT classifier operating on a graph of selected features. On the GDSC2 cohort of 2614 cell line–drug pairs, this pipeline reached an accuracy and F_1_-score of 95.87% and 95.87%, respectively, with AUROC and AUPRC values of 0.957 and 0.946. Under identical cross-validation splits, these results were consistently better than those obtained with conventional GAT, GNN, CNN, and DNN models. In addition, PPV, NPV, TPR, and TNR were all higher for EA-GAT, indicating that the improvement is not restricted to a single metric.

The ablation study allows us to better understand what contributes to the improvements made. By removing SKOA or MNJA, we see an approximate drop of about 1.7–1.9% in terms of accuracy and F_1_, and also some reduction in AUROC and AUPRC. Both the Smoluchowski-guided search for compact feature subsets and the topology-aware sequence representation, therefore, seem to be contributing more than just small refinements. Also, removing drug-structure descriptors or replacing the graph head with a non-graph classifier results in much greater loss of performance. This implies that the model benefits from having knowledge of the chemical structure of the drug and of the cellular environment (cell-line), and also from being able to represent dependencies between features, as opposed to representing each feature as an independent variable. Finally, while the contribution of the Aranda activation appears to be relatively minor, it does have some measurable impact; specifically, when removed from the model, there is a noticeable decline in all metric values, and the model takes longer to train. This is consistent with the reduced training time observed for EA-GAT.

Finally, if we examine the biological relevance of the model’s predictions, the most important question is whether the model’s ability to make accurate predictions can be attributed to plausible biochemical mechanisms or simply to correlation. There are several arguments supporting the idea that plausible biochemical mechanisms are at play. For example, the SHAP analysis shows that many high-ranking features correspond to well-known cancer-driving mutations/genes, including those involved in PI3K/AKT/mTOR signaling pathways, receptor tyrosine kinases, and regulation of cell cycle and apoptosis. Furthermore, when examining explanations produced for particular drug classes, we find that the top-ranking features corresponding to that drug class typically reflect known mechanism-of-action relationships. That is, PI3K pathway members are commonly among the highest-ranking features for PI3K inhibitors, mitotic and spindle-related genes are often among the top features for microtubule-targeting agents, and DNA repair/p53-related genes are frequently among the highest-ranking features for DNA-damaging drugs. Moreover, performing pathway enrichment on SHAP-ranked genes will recover the typical cascade signaling pathways used in tumor biology and treatment response.

The *in vitro* Pictilisib validation provides experimental confirmation of this interpretation. Across six cell lines representing diverse PI3K pathway contexts, the model achieved 83.3% binary classification accuracy and strong rank-order agreement (Spearman *ρ* = -0.89, p = 0.018) between predicted sensitivity scores and measured IC_50_ values. Response patterns matched known PI3K biology: PIK3CA-mutant lines (MCF7, T-47D, BT-474) exhibited IC_50_ values of 0.3–0.5 µM, consistent with pathway dependence, while PTEN-loss and KRAS-mutant lines (MDA-MB-231, A549) were resistant, with IC_50_ exceeding 2.5 µM. These values align with published Pictilisib data for the same lines. HCC1954, carrying both PIK3CA mutation and HER2 amplification, showed borderline response (IC_50_ = 1.12 µM) with an EA-GAT score of 0.61, appropriately reflecting the mechanistic complexity introduced by HER2-mediated bypass signaling. The validation thus supports, orthogonally, the EA-GAT predictions’ alignment with measurable variations in drug response, as well as the SHAP-derived feature consistency with previously reported PI3K inhibitor pharmacology. While compared to other published computational models that report a low level of *in-vitro* validation, i.e., Lee et al. 75% accurate on four cell lines, Chawla et al. 80% on five cell lines, Tang and Gottlieb 83% on six cell lines (*ρ* = 0.88), the results of the current study demonstrate comparable performance as well as provide gene- and pathway-level interpretations. Although the challenge in validating drug response predictive models across different data sets is significant, as shown by (26, 27), there were limited reductions in validated performance in the current study relative to cross-validation. Therefore, this reduction should be viewed cautiously due to the differences in validation panels, datasets, and protocols used across studies (26). More cross-dataset and experimental testing is required before the findings can be generalized.

The study has some limitations. First, all analyses were performed *in-vitro* and do not account for the tumor microenvironment, the innate biological and subclonal genetic variability of a tumor due to outpacing of pre-existing subclones, or prior exposure to other chemotherapeutic agents a patient may have received. Although predictive performance in these *in vitro* models does not directly translate to patient-derived disease models, validation will be an important subsequent aim. Second, the experimental validation is also limited in scope, since it involves only one drug and six cell lines. This phenomenon is also observed in other genes and with other drugs in fewer or shorter-term screens, and in larger panels of cell lines, but it is clearly worthy of investigation in more physiologically relevant models, including organoids and xenografts.

While we acknowledge that our results have limitations regarding response labels, we also see them as common challenges within the research community. In particular, by transforming continuous IC_50_ values into binary classifications in GDSC2, we established classification thresholds. Thus, cases on the borderline between the classes may be influenced by random error in the measurements used to create the classes. As noted by researchers in their review of recent benchmarking studies using cross-validation for modeling drug responses (26, 27), the best performance observed in cross-validation studies was frequently unstable when tested experimentally or in clinically relevant applications. Therefore, our 83.3% experimental accuracy is encouraging, but it should still be read as preliminary rather than definitive evidence of broader generalization.

As with the response labels, there are boundaries in terms of what SHAP can reveal. SHAP provides insights into relationships that the model found through data analysis; however, SHAP cannot identify causal relationships. To assess whether some of the top-ranked features, based on their SHAP scores, represent biologically relevant mechanisms involved in drug response, experiments manipulating the expression levels of the corresponding genes via CRISPR screening would be necessary. Similarly, the molecular input space is limited because proteome representation in the GDSC2 dataset is less informative than potentially informative representations such as epigenomes and metabolomes. Moreover, dynamic post-translational regulation, specifically phosphorylation, is not included. Finally, since the current framework only accounts for single-agent response at a fixed time point, multi-drug combination treatment and temporal response patterns are not accounted for in the current framework. Lastly, although we did not evaluate uncertainty quantification in detail, having a means to provide calibrated predictive intervals could be useful for decision-making based on predictions from downstream systems.

Although the approach has a few constraints, the findings provide evidence that multi-omics integration, graph-based learning, and *post-hoc* explanation can be integrated into a single system without compromising biological interpretability. While the experimental validation does not eliminate uncertainty, it reduces the likelihood that the model is simply dependent on superficial correlations. More generally, this study is not limited to data from GDSC2 and may be applied to other similar studies (such as how patients respond to immunotherapies or how sensitive they are to certain combination therapies), assuming there are sufficient multimodal data sets for the application.

## 5 Conclusion

We have developed an explainable AI framework for cancer drug response prediction that brings together several complementary ideas: MNJA-derived sequence trees to encode local sequence patterns, DS-GoogLeNet and handcrafted features to represent both genes and drugs, SKOA for selecting compact and informative feature subsets, and an EA-GAT classifier to exploit dependencies among features. Applied to the GDSC2 dataset, this combination achieved high predictive performance, with accuracy and F_1_-score of 95.87% and 95.87% and AUROC/AUPRC of 0.957 and 0.946, and outperformed a set of strong neural baselines under stratified 10-fold cross-validation.

Equally important, the framework yields explanations that can be interpreted in familiar biological terms. SHAP-based attributions highlight genes and drug-structure descriptors that are consistent with known mechanisms of action, and pathway enrichment of the most influential genes recovers PI3K–AKT, mTOR, MAPK, and related pathways that play central roles in tumor growth and therapy response. *In vitro* validation with Pictilisib on six cell lines demonstrated 83.3% binary accuracy and Spearman *ρ* = −0.89, confirming that predictions align with experimentally measured drug sensitivity and that SHAP-derived mechanistic interpretations reflect established PI3K pathway biology. In this way, the model functions not only as a predictor but also as a tool for hypothesis generation and biomarker prioritization.

Future studies should evaluate the proposed framework across a broader range of drugs, mechanisms of action, patient-derived xenograft models, organoids, and clinical cohorts with matched treatment and outcome information. Incorporating comprehensive phosphoproteomic measurements, uncertainty quantification, prediction of drug-combination effects, and modeling of resistance trajectories would further broaden the applicability of the method. In addition, perturbation-based experiments will be necessary to determine whether the top-ranked predictive features identified by the model have direct causal roles in cellular drug response.

## Data Availability

The original contributions presented in the study are included in the article/supplementary material. Further inquiries can be directed to the corresponding author.
